# One‐Step, High‐Removal‐Rate and Low‐Damage Chemical Mechanical Polishing of InP Enabled by Hydrolysis Activated PF_6_^−^ with In Situ Fluoride Passivation

**DOI:** 10.1002/advs.75142

**Published:** 2026-04-02

**Authors:** Shigong Fu, Jianwei Zhou, Chenwei Wang, Yanchao Ge, Chong Luo, Yuhang Qi

**Affiliations:** ^1^ School of Electronics and Information Engineering Hebei University of Technology Tianjin China; ^2^ Tianjin Key Laboratory of Electronic Materials and Devices Tianjin China

**Keywords:** ammonium hexafluorophosphate, chemical mechanical polishing, defect control, indium phosphide, quantum yield

## Abstract

Achieving high‐throughput, low‐damage preparation of indium phosphide (InP) surfaces remains challenging because increasing the material removal rate (MRR) in chemical mechanical polishing (CMP) often compromises ultralow roughness and requires post‐CMP passivation, which can lead to secondary etching. In this work, an ammonium hexafluorophosphate (NH_4_PF_6_) assisted CMP process is developed to incorporate directly into the polishing process via in situ fluoride passivation. Hexafluorophosphate (PF_6_^−^) undergoes In^3^^+^‐catalyzed hydrolysis, continuously producing fluoride‐rich intermediates that rapidly fluorinate InP, suppress oxide/defect formation, and concurrently generate an InF_3_‐rich, mechanically compliant reaction layer that is readily stripped by SiO_2_ abrasives; partial dissolution to soluble hexafluoroindate ([InF_6_]^3^^−^) further removes reaction products and sustains fast chemical–mechanical turnover. This approach achieves an MRR of 424 nm min^−1^ with a root‐mean‐square roughness (Sq) of 0.0862 nm. The fluoride‐passivated surfaces show improved optical quality, including longer time‐resolved photoluminescence (TRPL) lifetimes, higher photoluminescence quantum yields (PLQY), and more uniform emission, underscoring the promise of in situ fluoride passivation for scalable, device‐relevant InP surface preparation.

## Graphical abstract

In this work, we propose a CMP method for InP using NH_4_PF₆, which hydrolyzes under In^3+^ catalysis to form reactive species. These convert InP into low‐binding‐energy fluorides, facilitating removal and dissolution. The process delivers high material removal rates, smooth surfaces, and fluorine passivation that lowers defect density and improves optoelectronic properties.

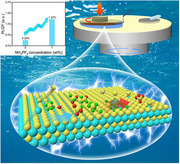

## 1 Introduction

Indium phosphide (InP) is a prototypical III–V direct‐bandgap semiconductor (E_g_ ≈ 1.35 eV) that underpins high‐performance optoelectronics and integrated photonics owing to its favorable carrier transport and electro‐optical properties [1, 2, 3, 4, 5, 6]. As device dimensions continue to shrink, atomic‐level control of surface and interface quality becomes increasingly critical for yield and reliability [7, 8]. However, wafer preparation and subsequent processing can introduce native oxides, scratches, and subsurface damage [9, 10, 11], which increase surface recombination and dark current, reduce luminescence and external quantum efficiency, and deteriorate carrier mobility [12, 13, 14]. Developing scalable approaches that simultaneously remove the damaged layer and suppress oxide‐ and defect‐related surface states remains a key prerequisite for next‐generation InP devices.

Chemical mechanical polishing (CMP) is widely adopted to remove damage layers and achieve near‐atomic planarization [15, 16, 17, 18, 19], but achieving a high material removal rate (MRR) with sub‐0.1 nm roughness and low defect density remains challenging. Conventional oxidative or halogen‐based chemistries can increase MRR but frequently introduce pitting, pad wear, or scratch defects. At the same time, many formulations that deliver ultralow roughness are restricted to small‐area samples or suffer from insufficient throughput [20, 21, 22, 23, 24, 25]. These limitations highlight that an effective CMP chemistry must not only promote controlled oxidation/complexation but also regulate interfacial reactivity and byproduct removal to avoid defect generation during high‐rate polishing.

Beyond morphology, post‐polishing InP surfaces commonly retain residual oxides and electronically active defects (dangling bonds and trap states), which elevate interface‐state density and contact resistance [26]. As a result, post‐CMP cleaning/passivation (e.g., sulfide treatments) is routinely employed to remove oxides and inhibit reoxidation [27, 28, 29, 30]. Nevertheless, these wet‐chemical steps can cause additional etching and partially offset the planarization achieved by CMP [31]. A CMP strategy that couples rapid planarization with concurrent, morphology‐preserving surface stabilization is therefore highly desirable.

Herein, we demonstrate an ammonium hexafluorophosphate (NH_4_PF_6_) assisted CMP route that couples high‐throughput planarization with in situ fluoride passivation. During polishing, PF_6_^−^ undergoes In^3^^+^‐catalyzed hydrolysis to continuously generate reactive fluoride‐containing intermediates, which fluorinate InP surfaces, suppress oxide regrowth, and mitigate defect formation. Moreover, this hydrolysis‐activated fluoride sustains a fast chemical mechanical removal cycle by forming a fluorinated reaction layer with weakened interfacial strength that is readily stripped and renewed under SiO_2_ abrasive action, thereby enabling a high MRR of 424 nm min^−^^1^ while preserving atomic‐scale smoothness (root‐mean‐square roughness, Sq = 0.0862 nm). The resulting fluoride‐terminated wafers exhibit improved photophysical behavior, including longer time‐resolved photoluminescence (TRPL) lifetimes, higher photoluminescence quantum yields (PLQY), and enhanced wafer‐scale emission uniformity.

## 2 Results and Discussion

**FIGURE 1:**
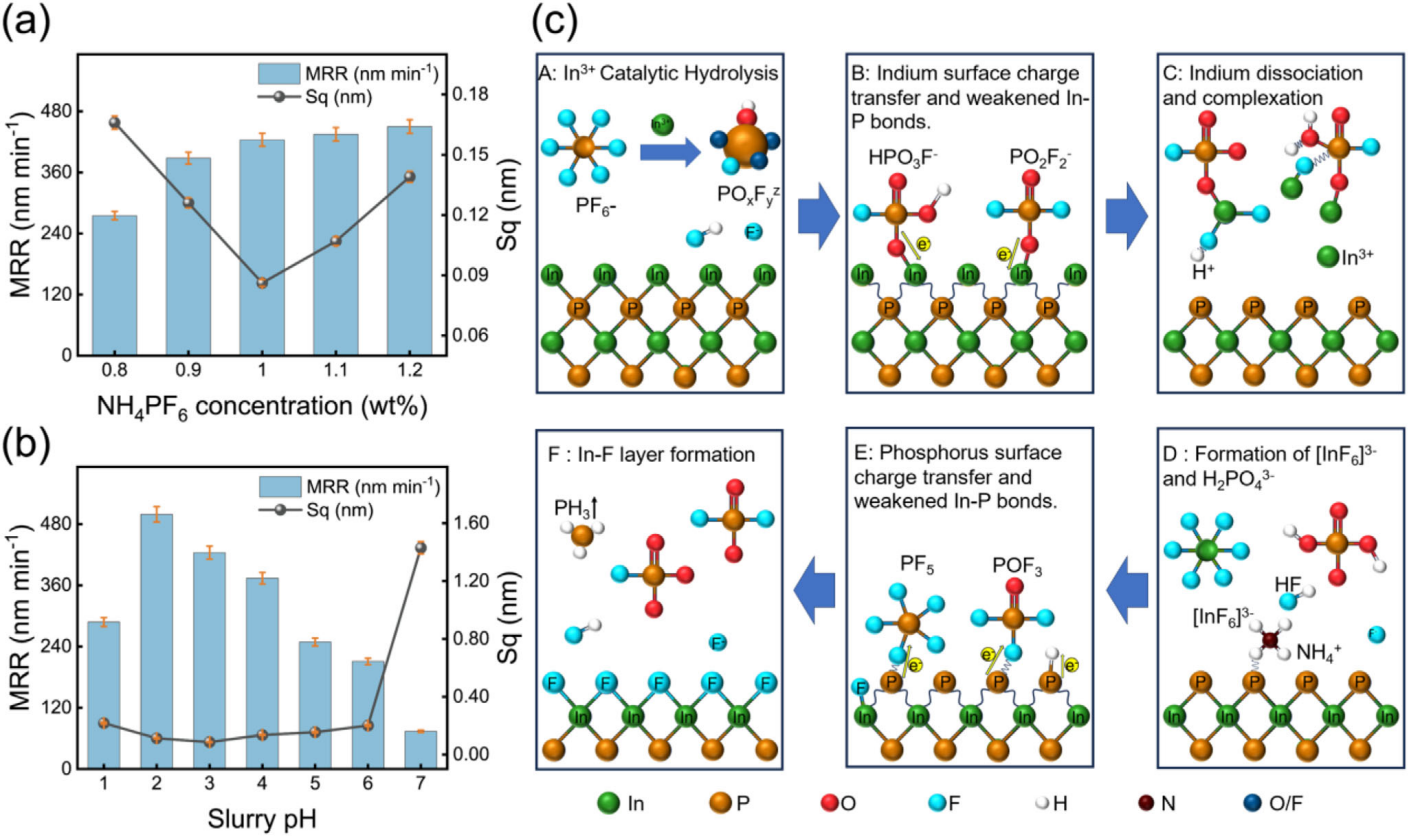
Dependence of the InP MRR and Sq on CMP parameters: (a) NH_4_PF_6_ concentration, and (b) slurry pH. (c) proposed reaction pathway of NH_4_PF_6_ with InP during CMP.

MRR and Sq were used to map the process window for InP CMP [32]. Both metrics depend on slurry composition and operating conditions, which also dictate defect formation (scratches, corrosion pits, and particle redeposition). The process parameters summarized in Figure S1 were systematically explored to evaluate the efficacy of NH_4_PF_6_ as a CMP additive. Atomic force microscopy (AFM) images are shown in Figures S2–S10, which led to the identification of the optimal conditions. Chemical reactivity was governed by the combined effects of NH_4_PF_6_ concentration and pH, which modulate the speciation of hydrolysis intermediates and their surface interactions (Figure 1a,b). The optimal parameters were identified as pH 3 and 1 wt.% NH_4_PF_6_, yielding a material removal rate (MRR) of 424 nm min^−^^1^ and a root‐mean‐square roughness (Sq) of 0.0862 nm [7, 33].

Having confirmed the superior CMP performance of the NH_4_PF_6_‐based slurry, it is important to clarify the chemical and physical mechanisms behind these results. Therefore, we propose the NH_4_PF_6_‐assisted CMP mechanism shown in Figure 1c. SiO_2_ abrasion first removes the native oxide, exposing fresh InP sites. PF_6_^−^ then undergoes stepwise hydrolysis at the interface, generating fluoride‐containing intermediates that fluorinate InP and form an InF_3_ surface layer. Relative to covalent InP, InF_3_ is more loosely packed and softer, making it readily removed by mechanical abrasion. Under acidic conditions, a fraction of InF_3_ dissolves to soluble hexafluoroindate species ([InF_6_]^3^^−^), which further accelerates the removal of fluoride reaction products and sustains the polishing cycle.

As polishing proceeds, dissolution of In^3^^+^ sustains PF_6_^−^ hydrolysis, maintaining a steady supply of reactive intermediates and continuous surface fluorination. The slurry remains chemically active throughout CMP, enabling efficient material removal and a uniformly smooth surface. During the reaction, phosphorus released from the In─P coordination can be partially protonated under acidic conditions and evolve as PH_3_; a smaller fraction reacts with dissolved oxygen or phosphorus‐oxide complexing agents to form soluble PO_x_ species, which are removed with the slurry. After CMP, residual PF^−^/F^−^ can form In─PF and In─F bonds that passivate dangling P bonds and suppress reoxidation of In and P, thereby reducing oxide‐related trap states and enhancing optoelectronic performance.

**FIGURE 2:**
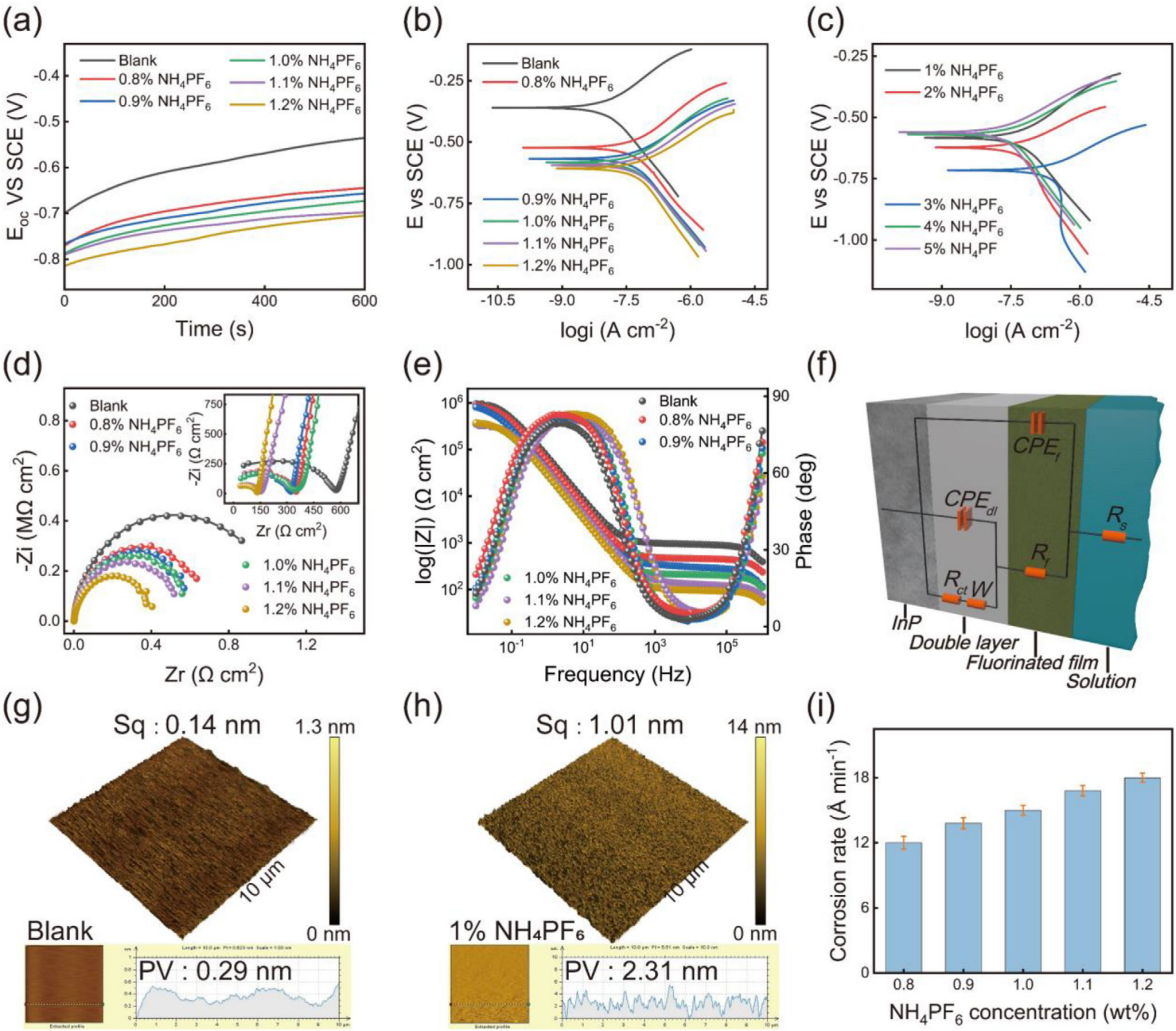
Electrochemical characterization of InP electrodes in NH_4_PF_6_ aqueous solutions: (a) OCP curves, (b) Tafel plots (0.8–1.2 wt.%), (c) Tafel plots (1–5 wt.%), (d) Bode plots, (e) Nyquist plots, and (f) equivalent circuit model used for EIS fitting. AFM images: (g) untreated InP, (h) InP after immersion in NH_4_PF_6_ solution. (i) Static corrosion rates.

To decouple polishing behavior from purely mechanical effects, we quantified corrosion/passivation of InP in NH_4_PF_6_‐containing solutions using open‐circuit potential (OCP)/Tafel analysis, static corrosion rates, and AFM characterization. As NH_4_PF_6_ increased from 0.8 to 3 wt.%, E_corr_ shifted to more negative values, and J_corr_ increased (Figure 2a,b; Table S1), indicating accelerated dissolution in the slurry. Increasing the concentration to 5 wt.% reversed this trend (more positive E_corr_ and lower J_corr_; Figure 2c), consistent with the formation of a reaction‐product film, a fluoride/oxyfluoride‐rich layer, that partially passivates the surface and suppresses further corrosion reactions [34]. However, due to the inherent high fluid shear forces and significant pressures in the CMP process, the passivation behavior observed under static conditions cannot be directly translated to the dynamic CMP interface. The mechanical action of the polishing pad and abrasives during CMP removes the passive film rapidly, preventing its conversion into a denser, more stable, and transpassive layer. Simultaneously, with constant mechanical action, the material removal rate of InP exhibited a saturation trend as the NH_4_PF_6_ concentration increased, a phenomenon attributed to mass transport (Figure S11).

Electrochemical impedance spectroscopy (EIS) further reveals concentration‐dependent interfacial evolution over 0.5–1.2 wt.% NH_4_PF_6_ (Figure 2d,e). Fits using the equivalent circuit (Figure 2f) yielded the parameters in Table S2. Increasing NH_4_PF_6_ from 0.8 to 1.2 wt.% lowered solution resistance (R_s_) from 8.25 to 3.80 Ω (Table S3), consistent with enhanced ionic conductivity. In parallel, the film resistance (R_f_) increased from 1.6 to 2.5 kΩ, and film capacitance (CPE_f_) increased from 0.25 to 0.40 µF, indicating rapid formation and densification of an InF_3_‐rich surface layer driven by PF_6_^−^ hydrolysis products that locally fluorinate/disrupt In─P bonds [35]. Thickening/compaction of the InF_3_ film impedes electron transfer across the InP/electrolyte interface, while its high dielectric constant and corrosion‐induced roughening increase the effective interfacial capacitance [36].

In contrast, the charge‐transfer resistance (R_ct_) decreased from 3.5 to 1.9 kΩ. At the same time, double‐layer capacitance (CPE_ct_) increased from 0.12 to 0.20 µF, consistent with enhanced charge transfer associated with fluorine adsorption and increased effective interfacial area during film formation [37]. The Warburg coefficient (W) increased from 1.4 to 5.3 Ω·s^−^^1^^/^^2^, reflecting slower ionic transport through the compact passivation layer at higher F^−^ coverage. AFM corroborated the strong chemical etching by NH_4_PF_6_: the surface remained smooth in deionized water (Figure 2g), whereas NH_4_PF_6_ yielded the highest roughness (Sq = 1.01 nm; PV = 2.31 nm; Figure 2h). Static corrosion rates followed the electrochemical trends and increased with NH_4_PF_6_ concentration in the low‐concentration regime (Figure 2i).

**FIGURE 3:**
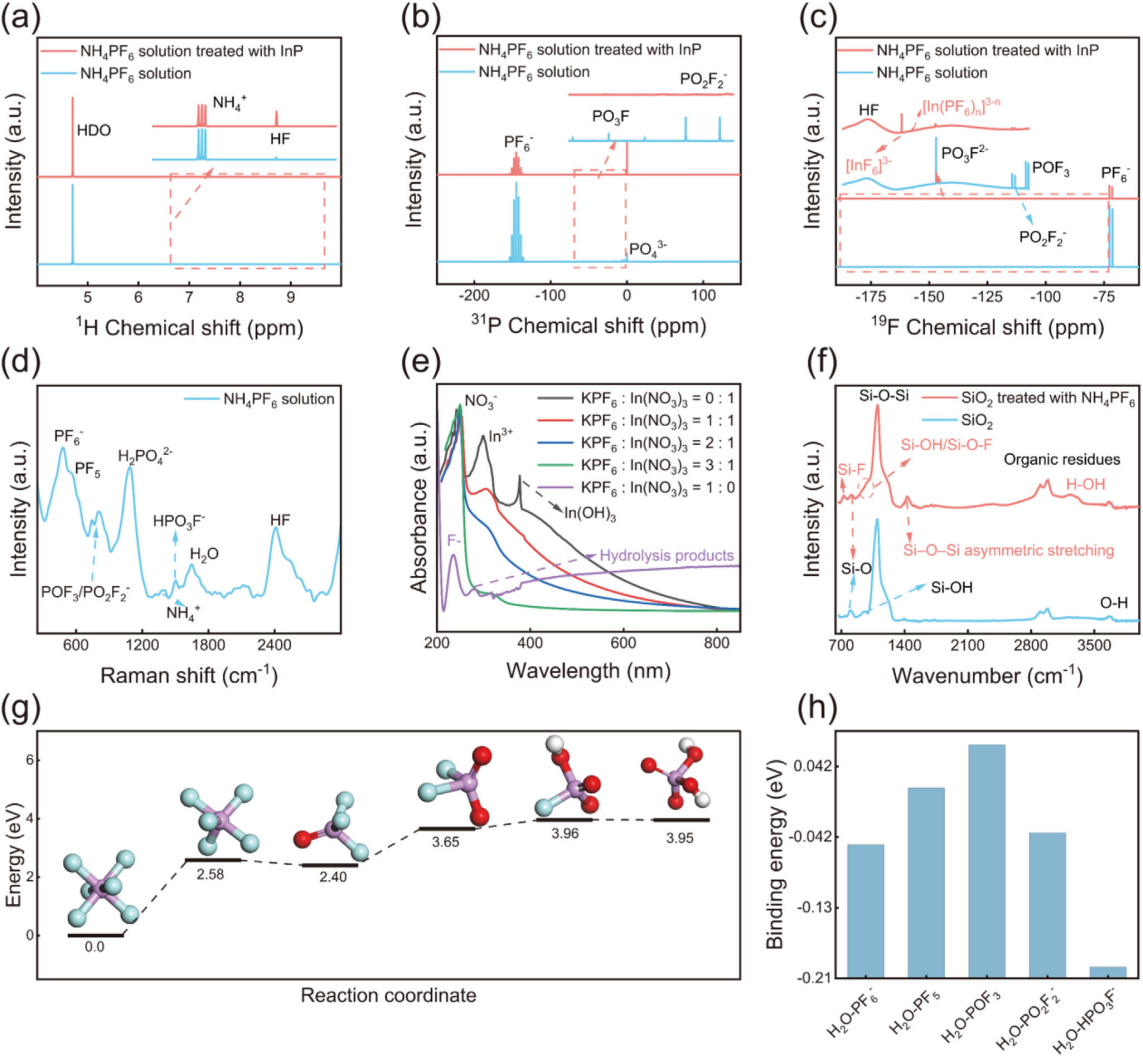
Spectroscopic analysis and proposed hydrolysis mechanism of NH_4_PF_6_ in contact with InP. NH_4_PF_6_ was dissolved in D_2_O, and InP was immersed for 5 min before NMR measurements: (a) ^3^ ^1^P NMR, (b) ^1^ ^9^F NMR, and (c) ^1^H NMR. (d) Raman spectrum of 1 wt.% NH_4_PF_6_ at pH 3. (e) UV–vis spectra of In(NO_3_)_3_/KPF_6_ mixtures with varying ratios. (f) FTIR spectra of SiO_2_ abrasives before/after exposure to NH_4_PF_6_ solution. (g) Schematic of sequential NH_4_PF_6_ hydrolysis. (h) Binding energies between ions/molecules and water.

To identify the solution speciation of NH_4_PF_6_ at pH 3 and its reaction pathway with InP, we combined liquid‐state nuclear magnetic resonance (NMR) and Raman spectroscopy. The data indicate that PF_6_^−^ undergoes P─F bond cleavage promoted by aqueous nucleophiles (H_2_O/OH^−^) and protons generated from NH_4_^+^ hydrolysis [38]. ^3^ ^1^P and ^1^ ^9^F NMR reveal progressive formation of H_2_PO_4_^−^, HPO_3_F^−^, PO_2_F_2_^−^, hydrofluoric acid (HF), and POF_3_ (Figure 3a–c). Introducing InP reduced the ^1^H signal of NH_4_^+^, consistent with electrostatic adsorption at the surface. It consumed HPO_3_F^−^/PO_2_F_2_^−^/POF_3_ while increasing HF and H_2_PO_4_^−^, implying that these intermediates directly react with InP to yield HF/H_2_PO_4_^−^ as dominant products. Notably, ^1^ ^9^F NMR shows soluble indium‐fluoride complexes ([InF_6_]^3^^−^ and [In(PF_6_)]^3^^−^), evidencing partial complexation of In^3^^+^ in NH_4_PF_6_ solutions [39, 40]. Raman spectroscopy further confirms dissociated NH_4_^+^ and PF_6_^−^ and the coexistence of hydrolysis products (H_2_PO_4_^−^, HPO_3_F^−^, PO_2_F_2_^−^, PF_5_, HF, and POF_3_; Figure 3d).

Contact‐angle and surface‐tension measurements show that NH_4_PF_6_ hydrolysis modulates slurry wetting and reaction kinetics during CMP. At higher pH, hydrolysis is suppressed, increasing both contact angle and surface tension, limiting slurry spreading and slowing surface reactions (Figure S12). Increasing NH_4_PF_6_ promotes hydrolysis, generating HF and other acidic species that lower the local pH, decrease contact angles and surface tension, improve wetting, and enable more uniform slurry coverage (Figure S13) [39]. However, excess NH_4_PF_6_ over‐accelerates chemical etching and induces surface roughening.

To probe the role of In^3^^+^ in NH_4_PF_6_ hydrolysis and InP removal, we performed NMR on NH_4_PF_6_ solutions containing In(NO_3_)_3_ (Figure S14). Adding In^3^^+^ promoted PF_6_^−^ hydrolysis, increasing POF_3_ and HPO_3_F^−^ and decreasing PF_6_^−^, while PO_2_F_2_^−^ remained nearly unchanged. The strong Lewis acidity of excess In^3^^+^ polarizes coordinated water, enhancing nucleophilic attack of H_2_O/OH^−^ on P─F bonds [41]. The resulting F^−^ readily replaces weakly bound PF_6_^−^ ligands in indium complexes [42], consistent with the predominance of soluble [InF_6_]^3^^−^ in the NMR spectra. UV–vis spectroscopy further indicates that In^3^^+^ hydrolyzes to In(OH)_3_ at pH 3 (Figure 3e); upon introducing PF_6_^−^, the In^3^^+^/In(OH)_3_ signatures diminish, and the solution turns from milky to transparent, consistent with soluble In─F or In─PF_6_ complexation (Figure S15). This complexation suppresses indium hydroxide precipitation on the wafer during polishing, thereby improving surface quality.

FTIR spectra of SiO_2_ abrasives after exposure to NH_4_PF_6_ (Figure 3f) suggest that trace HF generated from PF_6_^−^ hydrolysis etches the silica surface, partially converting Si─OH to Si─F and Si─O─F and exposing the underlying Si─O─Si network [43]. This surface fluorination can smooth particle morphology without altering the bulk silica lattice (Figure S16), potentially reducing the propensity for scratches. Fluorination also increases particle hydrophobicity, which can enhance mobility and promote a more uniform abrasive distribution [44]. However, as time passes, the hydrolysis of NH_4_PF_6_ increases. The resulting excess fluorination and changes in ionic strength compress the silica electrical double layer, promoting aggregation and decreasing polishing performance (Figures S17–S19) [45].

Based on these results, we propose the NH_4_PF_6_ hydrolysis sequence summarized in Figure 3g. Thermodynamic analysis indicates that PF_6_^−^ → PF_5_, POF_3_ → PO_2_F_2_^−^, and PO_2_F_2_^−^ → HPO_3_F^−^ are endergonic, whereas PF_5_ → POF_3_ and HPO_3_F^−^ → H_2_PO_4_^−^ are energetically favorable. Consistently, binding‐energy calculations show positive binding energy (E_bind_) for PF_6_^−^–H_2_O and POF_3_–H_2_O (weak association and low direct‐hydrolysis propensity). In contrast, other intermediates exhibit negative E_bind_ and thus stronger interactions with water (Figure 3h). Collectively, these results indicate that PF_6_^−^ is thermodynamically stable and hydrolyzes only under catalysis or external‐field perturbation. Once intermediates such as PF_5_ or HPO_3_F^−^ form, subsequent steps become spontaneous and proceed readily. Accordingly, NH_4_PF_6_ remains largely stable in the absence of polishing, supporting slurry shelf stability. In contrast, the hydrolysis of PF_6_^−^ is catalyzed by dissolved In^3^^+^ to generate reactive species that accelerate InP removal during CMP. In parallel, NH_4_^+^ buffers the local pH, mitigating rate fluctuations as reactions progress and enabling sustained chemical activity during polishing.

**FIGURE 4:**
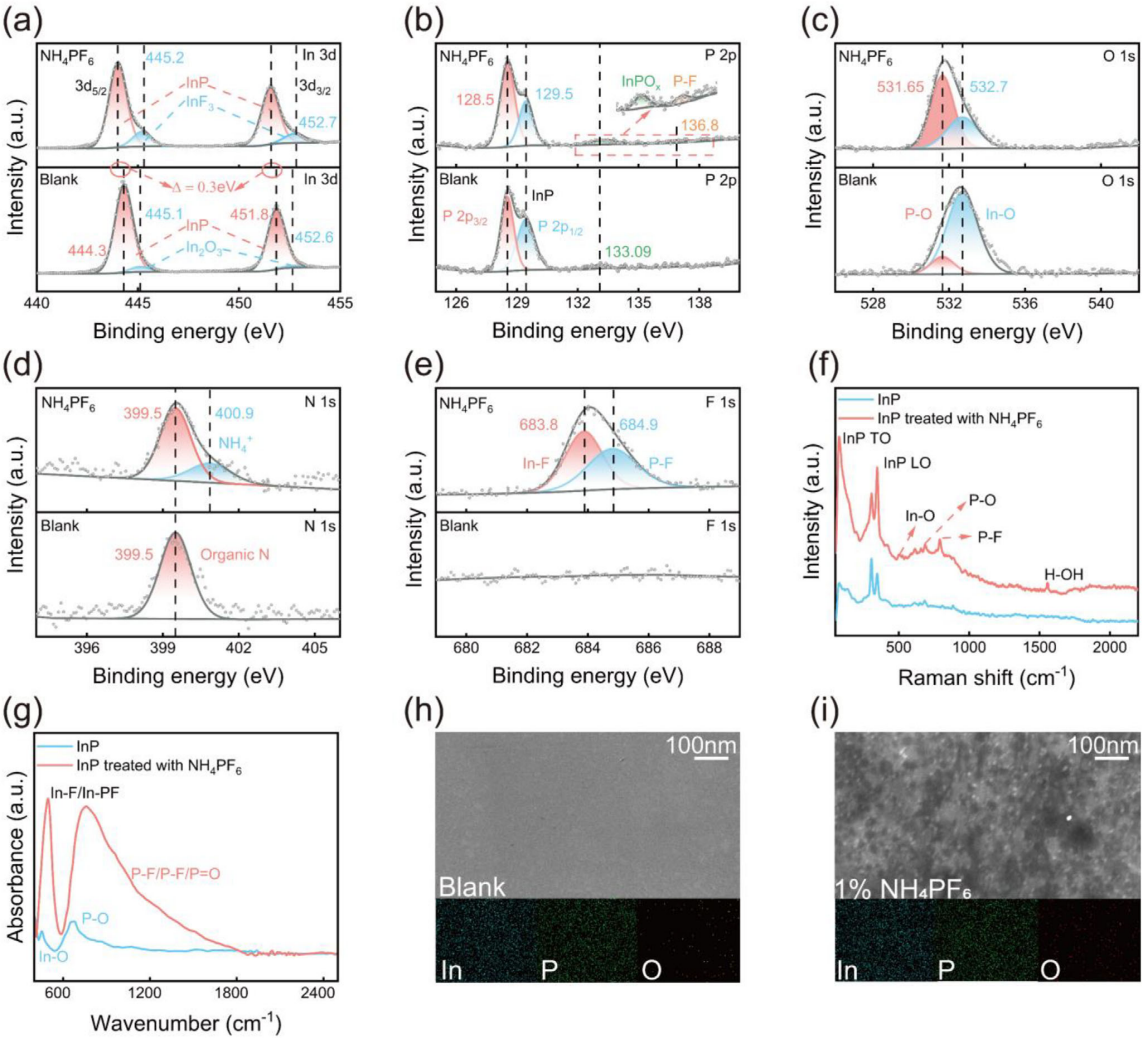
Surface and materials characterization of InP before and after NH_4_PF_6_ immersion treatment. XPS spectra: (a) In 3d, (b) P 2p, (c) O 1s, (d) N 1s, and (e) F 1s. (f) Raman spectra. (g) FTIR spectra. SEM images: (h) untreated wafer and (i) wafer after immersion in NH_4_PF_6_ solution.

We next examined changes in the InP surface chemistry after exposure to NH_4_PF_6_ by X‐ray photoelectron spectroscopy (XPS) (Figure 4). For the untreated wafer, the In 3d_3_⁄_2_/In 3d_5_⁄_2_ doublet appears at ∼451.8/444.3 eV (Figure 4a) and the P 2p doublet at 128.53/129.50 eV (Figure 4b). Minor oxidation produces additional In─O components (445.1 and 452.6 eV; In_2_O_3_/In(OH)_3_) [46] and a P─O component at 133.09 eV (InPO_4_/In(PO_3_)_3_) [47]. Quantitative fitting indicates ∼2.80% oxidized In and ∼4.85% oxidized phosphorus at the surface.

After NH_4_PF_6_ treatment, the In 3d peaks shift slightly to 451.5/444.0 eV, consistent with surface fluorination [48]. The In─O component at 445.1 eV is converted to indium fluoride, increasing the fluoride fraction to 15.12% (Figure 4a). Phosphorus‐containing species also increase to 7.17%, consistent with adsorption of phosphate/POF_3_ derived from PF_6_^−^ hydrolysis (Figure 4b). The O 1s fitting shows that the indium‐oxide‐to‐indium‐phosphate ratio decreases from ∼63:36 to ∼13:86, consistent with deposition of a fluorinated/phosphate‐rich surface film (Figure 4c). In the N 1s spectrum, a new peak at 399.5 eV is assigned to surface‐bound NH_4_^+^ (Figure 4d). The F 1s spectrum shows components at 683.79 and 685.95 eV, consistent with In─F and/or In─PF_6_ bonding (Figure 4e) [49]. The increased post‐treatment contact angle supports a more hydrophobic, fluorinated surface (Figure S20).

Raman spectroscopy shows that NH_4_PF_6_ treatment increases the intensities of the transverse optical (TO) and longitudinal optical (LO) phonon modes, with a more pronounced enhancement of the LO mode (Figure 4f). This trend is consistent with reduced surface defect density and increased near‐surface carrier density [32]. Although an In─F vibration is not clearly resolved because it is masked by strong InP phonon modes and overlapping P─O/F bands, a distinct feature at 791 cm^−^^1^ confirms P─F bonding and supports the presence of In─PF_6_‐related surface species. Weak In─O and P─O features are also observed, suggesting partial deposition/oxidation of reaction products at the surface.

FTIR further corroborates surface fluorination (Figure 4g). The untreated wafer exhibits weak bands at 450 and 660 cm^−^^1^, assigned to In─O and P─O vibrations. After NH_4_PF_6_ treatment, these features shift to ∼500 and ∼760 cm^−^^1^, consistent with In─F and P─F bonding. An extended tail near 760 cm^−^^1^ suggests deposition of PF_6_^−^ hydrolysis products and vibrational coupling among surface P─O, P═O, and P─F groups [7]. SEM imaging reveals the expected corrosive signature after immersion (Figure 4h,i). EDS shows substantial fluorine incorporation, providing direct evidence of fluorine passivation (Figure S21).

The corrosion mechanisms of various ionic species on InP were investigated by comparing solutions of NH_4_PF_6_, KPF_6_, NH_4_F, NH_4_H_2_PO_4_, and NH_4_H_2_PO_4_ + KF, all adjusted to a pH of 3 (Figure S22, Tables S4 and S5). NH_4_^+^ play two critical roles: they facilitate the dissolution of phosphate byproducts from the InP surface and buffer the local pH, thereby sustaining the hydrolysis of PF_6_^−^ and the continuous generation of reactive species [33]. Concurrently, NH_4_^+^ promotes the exposure of active indium sites necessary for forming In─PF_6_ surface complexes (Figure S23 and S24). The corrosion of InP by PF_6_^−^ is relatively mild. Its primary effect is indirect, mediated through the reaction of its hydrolysis products with the InP surface. The resulting fluoride ions (F^−^) are the principal agents of direct chemical corrosion [34]. This controlled process prevents significant alterations in the wafer surface topography (Figure S25).

To rationalize the reactivity of PF_6_^−^ hydrolysis products toward InP, we computed Fukui indices, electrostatic potential (ESP) maps, frontier orbital energies, and adsorption energies for representative species (Figure S26). The results indicate that PF_5_ and POF_3_ primarily act as electrophiles, targeting surface P atoms, facilitating In─P bond cleavage, exposing indium sites, and accelerating InF_3_ formation [38]. Oxygen‐containing species such as PO_2_F_2_^−^ and HPO_3_F^−^ are strongly nucleophilic toward In atoms (via O lone pairs), forming surface In─O─POF_2_ or In─O─HPO_2_F intermediates that can subsequently convert to In─F under HF or dissolve to yield soluble [InF_6_]^3^^−^ [35]. This coupled electrophilic‐on‐P and nucleophilic‐on‐In is consistent with the experimentally observed increase in MRR and concomitant reduction in surface defect density. Although PO_4_^3^^−^ exhibits appreciable adsorption energies, its high structural stability/coordination saturation limits direct participation in these reactions [39].

**FIGURE 5:**
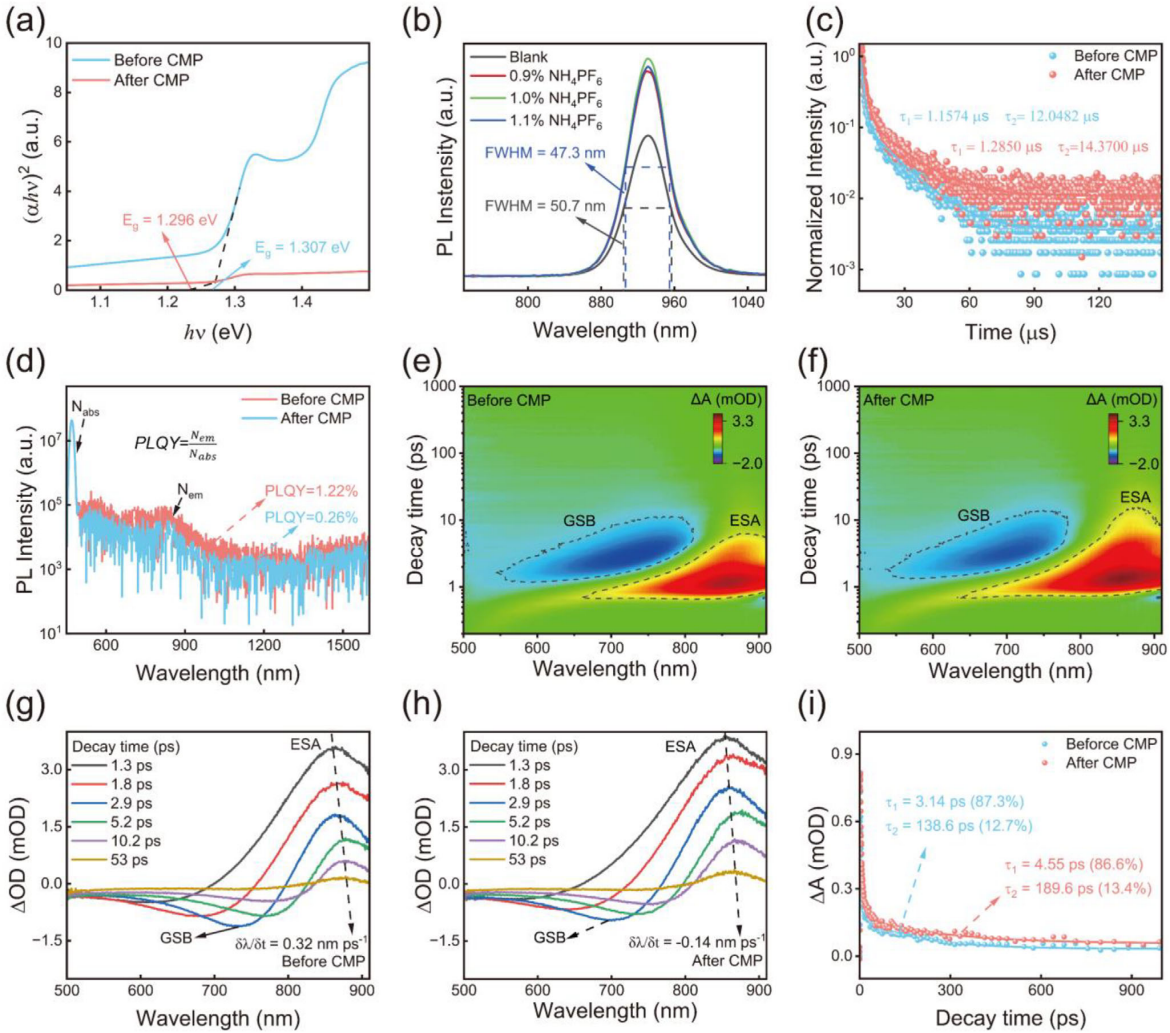
Defect characterization and ultrafast optical response of InP wafers before and after NH_4_PF_6_‐assisted CMP: (a) optical bandgap, (b) PL spectra at different NH_4_PF_6_ concentrations, (c) TRPL decay curves, and (d) PLQY. Transient absorption (400 nm excitation): ΔA maps of the original wafer (e) and NH_4_PF_6_‐CMP wafer (f); time evolution of ΔOD for the original wafer (g) and NH_4_PF_6_‐CMP wafer (h); (i) exciton‐decay kinetics in InP.

We next evaluated how NH_4_PF_6_‐assisted CMP enhances the optical properties and morphology of InP (Figure 5). The UV–vis absorption spectra indicate that the CMP process removed the severely damaged and disordered surface layer, exposing the underlying single‐crystal structure, which led to an overall reduction in absorption intensity. Subsequently, a mixed product layer of indium fluorophosphate, indium phosphate, and indium fluoride formed on the surface (Figure S27). Meanwhile, the UV–vis reflectance spectra reveal that the CMP treatment restored the reflective features associated with the intrinsic electronic structure of InP (Figure S28) [40]. X‐ray diffraction (XRD) likewise confirms removal of the surface damage layer (Figure S29).

Tauc analysis indicates that the pre‐CMP bandgap was 1.307 eV, a value that deviates from the intrinsic properties of InP (Figure 5a). This deviation suggests that extensive surface defects and stress induced the formation of band tail states extending from the valence band deep into the forbidden gap. These states enabled sub‐bandgap absorption, causing the apparent bandgap, extrapolated from the Tauc plot, to be reduced. After CMP, the damaged layer with its defects was removed from the InP wafer surface. The further reduction in bandgap (1.296 eV) signifies that the NH_4_PF_6_ CMP process induced surface electronic reconstruction [50]. This result suggests that the NH_4_PF_6_‐assisted CMP process suppresses deep‐level defect states while inducing a modified near‐surface electronic structure, possibly including states close to the band edge. Compared with deep‐level defects, such near‐band‐edge states are expected to be less detrimental to radiative recombination and may contribute to the enhanced luminescence and prolonged carrier lifetime observed after CMP.

Photoluminescence excitation spectra show that NH_4_PF_6_ treatment enhances emission while suppressing defect‐state absorption associated with surface oxides/vacancies (Figure S30) [51]. Correspondingly, photoluminescence (PL) spectra exhibit higher intensity with reduced non‐radiative recombination: both the full width at half maximum (FWHM) and the Stokes shift decrease (Table S6), and the extracted bandgap changes slightly from 1.333 to 1.332 eV (Figure 5b). These findings differ from conclusions drawn for fluorinated InP nanocrystals, where quantum confinement effects amplify surface electronic disorder. In contrast, bulk InP possesses a complete and long‐range ordered crystal lattice; thus, its measurements are predominantly influenced by the underlying lattice. Consequently, the fluorination‐induced disorder that is prominent in nanocrystals is much less pronounced in bulk InP wafers, which is consistent with the smaller Stokes shift observed here [52].

To assess the relative contributions of PO_x_ species and surface passivation to the fluorescence emission properties of InP, the PL intensities of samples treated with HF, H_2_O_2_, and a combined HF+H_2_O_2_ solution at identical pH were compared (Figure S31). The results indicate that the negative influence of increased surface PO_x_ species is weaker than the beneficial effect of fluoride passivation. Moreover, because the post‐CMP surface shows less pronounced residual PO_x_ features together with clear fluorine‐related passivation signals, the improved optoelectronic performance of NH_4_PF_6_‐treated InP is mainly attributed to fluoride passivation (Figure S32) [12].

To quantify suppression of non‐radiative recombination and exclude optical‐interference artefacts, we performed TRPL measurements (Figure 5c). After CMP, the fast component τ_1_ increases from 1.15 to 1.29 µs (≈11% reduction in surface‐trap‐mediated loss), and the slow component τ_2_ increases from 12.0 to 14.5 µs (≈21% improvement in bulk radiative recombination). The weighted average lifetime τavg increases from 10.64 to 12.30 µs, consistent with the enhanced PL intensity and reduced Stokes shift. The PL quantum yield increases from 0.26% to 1.22% (4.69×) after NH_4_PF_6_ polishing (Figure 5d), exceeding the 0.82% obtained with a commercial slurry and supporting effective fluorine passivation of surface defect states (Figure S33).

We further probed near‐surface carrier dynamics by femtosecond transient absorption spectroscopy (fs‐TAS) (Figure 5e,f). Under the Burstein–Moss effect associated with heavy sulfur doping, the ground‐state bleaching (ground‐state bleach (GSB), 500–800 nm) partially overlaps with ESA (600–900 nm). To minimize contributions from positive excited‐state absorption (ESA), kinetics were fit at 650 nm (Figure 5g,h). For the as‐received wafer, ESA decays rapidly, consistent with strong trapping/recombination, and the GSB peak exhibits a time‐dependent redshift indicative of fast carrier‐density loss and transient bandgap narrowing.

After NH_4_PF_6_‐assisted CMP, the ESA decay constant increases and the trap‐related fractional component decreases, indicating longer‐lived excited carriers and reduced trap density. Consistently, the GSB redshift is attenuated, and δλ/δt decreases, suggesting improved bandgap stability, slower carrier‐density evolution, reduced strain/band‐energy fluctuations, and diminished spectral broadening. Kinetic fitting reveals that after treatment with NH_4_PF_6_, the time constants corresponding to both trap state relaxation (τ_1_) and band‐edge carrier recombination (τ_2_) for InP are increased compared to the pristine sample. This indicates that surface defect states on the InP are effectively passivated, which reduces the probability of carrier capture by traps and allows more carriers to participate in the band‐edge recombination process. However, the relative amplitudes associated with τ_1_ and τ_2_ demonstrate that although the NH_4_PF_6_‐induced passivation reduces the defect states, defect‐assisted recombination remains the dominant pathway for carrier relaxation (Figure 5i).

**FIGURE 6:**
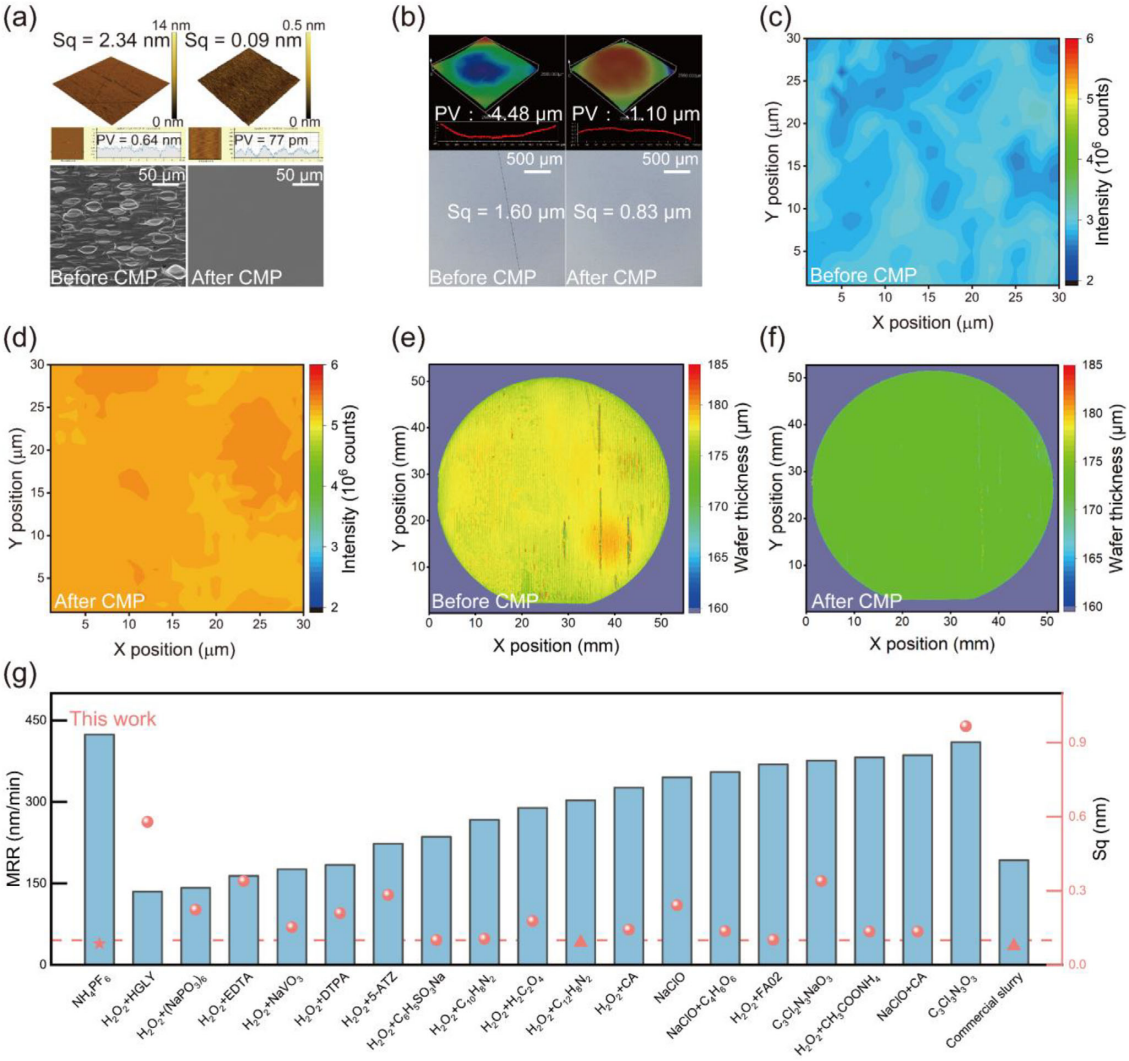
Surface morphology and optical uniformity of InP wafers before and after NH_4_PF_6_‐assisted CMP: (a) AFM and SEM images, (b) confocal microscopy, (c) PL mapping before polishing, (d) PL mapping after polishing, (e) 3D profilometry before polishing, (f) 3D profilometry after polishing, and (g) comparison of NH_4_PF_6_ with other additives for InP CMP.

Figure 6 summarizes the morphology and uniformity improvements enabled by NH_4_PF_6_‐assisted CMP. AFM shows that surface scratches are eliminated, with Sq reduced from 2.34 to 0.0862 nm and PV reduced to 77 pm. SEM confirms effective removal of the damaged layer and a smoother surface with fewer observable defects (Figure 6a). Confocal microscopy indicates a decrease in microscopic roughness from 1.550 to 0.826 µm (Figure 6b). PL mapping shows higher and more uniform emission: the average peak intensity increases from 82 800 to 92 000 counts, and the relative standard deviation decreases from 19.14% to 16.90% (Figure 6c,d). 3D profilometry yields an overall roughness of 2.5367 µm and flatness of 1.619 µm after CMP (Figure 6e,f).

Benchmarking against other polishing formulations shows that NH_4_PF_6_ delivers the highest MRR among the tested systems while maintaining excellent surface quality (Figure 6g). The resulting roughness is second only to a commercial InP fine‐polishing slurry (0.0767 nm) and meets the common industrial requirement (<0.1 nm). Although NH_4_PF_6_ yields a slightly higher Sq than the commercial slurry, its MRR is approximately doubled. Moreover, the TRPL lifetimes and PLQY improvements exceed those obtained with the commercial formulation (Figure S34). This enhancement suggests that the improved optical performance of InP following NH_4_PF_6_ CMP can be attributed to the combined effects of fluorination and the reduced PO_x_ concentration achieved by the CMP process.

## 3 Conclusion

In summary, an NH_4_PF_6_‐assisted CMP strategy is developed to enable concurrent high‐throughput planarization and in situ fluoride passivation of InP wafers. Under polishing conditions, In^3^^+^ catalyzes the hydrolysis of PF_6_^−^ to continuously generate fluoride‐rich intermediates, which promote rapid surface fluorination, regulate interfacial oxidation/etching, and suppress defect formation. This hydrolysis‐activated fluoride accelerates material removal by establishing a fluorinated reaction layer with reduced interfacial strength that can be readily refreshed and stripped under SiO_2_ abrasive action; the concurrent formation of soluble fluoroindate complexes further facilitates product removal and sustains fast chemical–mechanical turnover. As a result, the optimized process delivers an MRR of 424 nm min^−^^1^ together with atomically smooth surfaces (Sq = 0.0862 nm) and minimized oxide residues. Fluoride termination passivates surface states, leading to longer carrier lifetimes, a 4.69‐fold enhancement in PLQY, and improved wafer‐scale optical uniformity. This fluorination‐assisted CMP concept offers a scalable route for preparing InP substrates with combined topographical and electronic quality, supporting advanced photonic and electronic device manufacturing.

## 4 Experimental Section

### 4.1 Materials

All solutions were prepared with deionized water of resistivity ≥ 18.2 MΩ·cm. Colloidal silica (solid content = 11.27 wt.%; Jinwei Group Co., Ltd., China) was used as the abrasive material. The chemical reagents employed included ammonium hexafluorophosphate, potassium hexafluorophosphate, ammonium fluoride, ammonium dihydrogen phosphate, potassium fluoride, indium nitrate, glycine, sodium hexametaphosphate, sodium metavanadate, ethylenediaminetetraacetic acid (EDTA), diethylenetriaminepentaacetic acid (DTPA), 5‐amino‐1,2,4‐triazole (5‐ATZ), sodium benzenesulfonate, 2,2'‐bipyridine, 1,10‐phenanthroline, oxalic acid, citric acid, tartaric acid, FA02, ammonium acetate, hydrogen peroxide, sodium hypochlorite, Hydrofluoric Acid, sodium dichloroisocyanurate, and trichloroisocyanuric acid. All reagents were purchased from Shanghai Yi'en Chemical Technology Co., Ltd. and used without further purification. A commercial InP final‐polishing slurry was obtained from Shanghai Xin'anna Electronic Technology Co., Ltd. Potassium hydroxide (KOH), nitric acid (HNO_3_), and sulfuric acid (H_2_SO_4_) were employed for pH adjustment of the polishing slurry. All comparative experiments were conducted at a pH of 3, and the concentrations of NO_3_^−^ and SO_4_^2^^−^ in the solution were maintained at identical levels. Sulfur‐doped n‐type InP wafers were used in the polishing experiments (diameter = 2 inches).

### 4.2 CMP Experiment

CMP experiments were performed on an RX 500 polisher equipped with a Politex polishing pad. Prior to each run, the pad surface was conditioned with a diamond dresser for 5 min to restore surface asperities and ensure uniform slurry distribution. Following polishing, wafers were rinsed thoroughly with deionized (DI) water and dried under nitrogen gas. The MRR was determined gravimetrically according to Equation (1) [53]:

(1) $$MRR=\frac{\Delta m}{\rho \pi R^{2}t}$$

where Δ*m* is the mass change before and after polishing, measured on an ME 204 E analytical balance (Mettler Toledo, Switzerland; readability = 0.1 mg), ρ is the density of InP (4.787 g·cm^−^^3^), *R* is the wafer's radius, and *t* is the polishing time. For each wafer, five gravimetric measurements were taken; the highest and lowest readings were discarded, and the mean of the remaining three was calculated. Every polishing condition was tested in triplicate, and the results were reported as the mean value.

### 4.3 Electrochemical Experiment

Electrochemical tests were carried out on a CHI 660 E workstation (CH Instruments, Shanghai, China) using a standard three‐electrode configuration. The InP wafers served as the working electrode, a saturated calomel electrode (SCE) as the reference, and a platinum electrode as the counter electrode. Before measurement, the InP electrodes were masked with adhesive tape to define an active area of 1 cm^2^. The exposed surface was polished with 2000‐grit silicon carbide paper to obtain a mirror finish, rinsed with deionized water (DI, resistivity ≥ 18.2 MΩ·cm), and dried under compressed air. The open‐circuit potential (*E*_oc_) was monitored for 600 s until stabilization. Potentiodynamic polarization curves were recorded over the range *E*_oc_ ± 0.30 V at a scan rate of 5 mV s^−^^1^. Electrochemical impedance spectroscopy (EIS) was performed at *E*_oc_ across the 0.01 Hz to 1 MHz frequency range, and the resulting data were analyzed using ZsimpWin software. Tafel plots were obtained by scanning at 10 mV s^−^^1^ over *E*_oc_ ± 300 mV.

### 4.4 Surface Characterization Experiment

Surface morphology and roughness before and after CMP were characterized using atomic force microscopy (AFM, Agilent 5600 LS, Agilent Technologies, USA) in tapping mode. Scanning electron microscopy (SEM, Sigma 500, Carl Zeiss, Germany) at 1000× magnification was used to observe morphological changes in InP wafers before and after immersion in polishing slurries with and without NH_4_PF_6_. Surface chemical composition was determined by X‐ray photoelectron spectroscopy (XPS, ESCALAB‐250Xi, Thermo Fisher Scientific, USA) using a Cu Kα X‐ray source (1486.6 eV). Spectra were calibrated to the C 1s peak at 284.8 eV and processed with Avantage software. Raman spectra were recorded on a Horiba Xplora Plus system. X‐ray diffraction (XRD) patterns were obtained using a Bruker AXS D8 Discover diffractometer. UV analyzed optical properties‐vis spectroscopy (Lambda 1050+, PerkinElmer, USA) over 200–1200 nm and photoluminescence (PL) spectroscopy (FLS 1000, Edinburgh Instruments, UK) with excitation from 500–1500 nm in 1 nm steps. Time‐resolved photoluminescence (TRPL) and PL quantum yield (PLQY) were measured using the Same Edinburgh FLS 1000 system. Confocal microscopy was performed with an Olympus LEXT‐OLS50‐SW. For chemical analysis in solution, NMR spectra of NH_4_PF_6_ aqueous solutions were collected at 500 MHz on a Bruker Avance NEO 600 spectrometer (Bruker, Germany) using D_2_O as solvent. FT‐IR spectra were obtained with a Thermo Scientific Nicolet 6700 spectrometer. Slurry particle size and zeta potential were measured using a laser nanoparticle size analyzer (PSS 380, Particle Sizing Systems, USA). Contact angle measurements were performed with a goniometer (Powereach JC2000D, China).

### 4.5 Quantum Chemical Calculations

Molecular dynamics (MD) simulations were performed using Materials Studio (BIOVIA, USA) to investigate the interactions between additive molecules and the InP (100) surface [54, 55]. Quantum‐chemical calculations were performed using the DMol3 and CASTEP modules. Geometry optimizations employed the generalized gradient approximation (GGA) with the Perdew–Burke–Ernzerhof (PBE) functional and a double numerical plus polarization (DNP) basis set. Fukui indices and HOMO–LUMO energies were determined to identify reactive sites on the surface and molecules. Adsorption studies were conducted using 4 × 4 InP(100) supercells comprising four atomic layers, with the bottom two layers fixed to simulate the bulk material. A vacuum spacing of 15 Å was applied normal to the surface to prevent artificial interactions between periodic images. Brillouin zone Sampling was performed with an 8 × 8 × 8 k‐point grid and a plane‐wave cutoff energy of 335 eV.

Adsorption energies (*E_ads_*) were calculated according to:

$$E_{ads}=E_{total}\;-\left(E_{molecule}+E_{substrate}\right)$$

where *E_total_* is the total energy of the adsorbate–substrate system, *E_molecule_* is the energy of the isolated molecule, and *E_substrate_* is the energy of the clean InP (100) surface [56].

## Funding

National Natural Science Foundation of China (52402246); the Natural Science Foundation of Hebei Province (E2023202267).

## Conflicts of Interest

The authors declare no conflicts of interest.

## Supporting information




**Supporting File**: advs75142‐sup‐0001‐SuppMat.docx.

## Data Availability

The data that support the findings of this study are available from the corresponding author upon reasonable request.

## References

[1] E. Jang and H. Jang , “Review: Quantum Dot Light‐Emitting Diodes,” Chemical Reviews 123 (2023): 4663–4692. 36795794 10.1021/acs.chemrev.2c00695

[2] J. Kim , J. Roh , M. Park , and C. Lee , “Recent Advances and Challenges of Colloidal Quantum Dot Light‐Emitting Diodes for Display Applications,” Advanced Materials 36 (2024): 2212220. 10.1002/adma.202212220 36853911

[3] G. Lihachev , J. Riemensberger , W. Weng , et al., “Low‐Noise Frequency‐Agile Photonic Integrated Lasers for Coherent Ranging,” Nature Communications 13 (2022): 3522. 10.1038/s41467-022-30911-6 PMC9209488 35725718

[4] P. Yu , S. Cao , Y. Shan , et al., “Highly Efficient Green InP‐Based Quantum Dot Light‐Emitting Diodes Regulated by Inner Alloyed Shell Component,” Light: Science & Applications 11 (2022): 162. 10.1038/s41377-022-00855-z PMC9151710 35637219

[5] K. Liang , R. Wang , B. Huo , et al., “Fully Printed Optoelectronic Synaptic Transistors Based on Quantum Dot–Metal Oxide Semiconductor Heterojunctions,” ACS Nano 16 (2022): 8651–8661. 35451308 10.1021/acsnano.2c00439

[6] Y. Bian , X. Yan , F. Chen , et al., “Efficient Green InP‐Based QD‐LED by Controlling Electron Injection and Leakage,” Nature 635 (2024): 854–859. 39567695 10.1038/s41586-024-08197-z

[7] F. Zhang , X. Zhang , Z. Li , et al., “A New Strategy for Selective Area Growth of Highly Uniform InGaAs/InP Multiple Quantum Well Nanowire Arrays for Optoelectronic Device Applications,” Advanced Functional Materials 32 (2022): 2103057.

[8] Z. Yan , Y. Han , L. Lin , et al., “A Monolithic InP/SOI Platform for Integrated Photonics,” Light: Science & Applications 10 (2021): 200. 10.1038/s41377-021-00636-0 PMC8473568 34565795

[9] H. Li , W. Zhang , Y. Bian , T. K. Ahn , H. Shen , and B. Ji , “ZnF_2_ ‐Assisted Synthesis of Highly Luminescent InP/ZnSe/ZnS Quantum Dots for Efficient and Stable Electroluminescence,” Nano Letters 22 (2022): 4067–4073. 35536635 10.1021/acs.nanolett.2c00763

[10] Q. Wu , F. Cao , S. Wang , et al., “Quasi‐Shell‐Growth Strategy Achieves Stable and Efficient Green InP Quantum Dot Light‐Emitting Diodes,” Advanced Science 9 (2022): 2200959. 35618484 10.1002/advs.202200959 PMC9313472

[11] H. Baek , S. Kang , J. Heo , et al., “Insights into Structural Defect Formation in Individual InP/ZnSe/ZnS Quantum Dots under UV Oxidation,” Nature Communications 15 (2024): 1671. 10.1038/s41467-024-45944-2 PMC10891109 38396037

[12] R. F. Ubbink , G. Almeida , H. Iziyi , et al., “A Water‐Free In Situ HF Treatment for Ultrabright InP Quantum Dots,” Chemistry of Materials 34 (2022): 10093–10103. 36439318 10.1021/acs.chemmater.2c02800 PMC9686131

[13] Y. Kim , S. Ham , H. Jang , et al., “Bright and Uniform Green Light Emitting InP/ZnSe/ZnS Quantum Dots for Wide Color Gamut Displays,” ACS Applied Nano Materials 2 (2019): 1496–1504.

[14] M. Stam , G. Almeida , R. F. Ubbink , et al., “Near‐Unity Photoluminescence Quantum Yield of Core‐Only InP Quantum Dots via a Simple Postsynthetic InF_3_ Treatment,” ACS Nano 18 (2024): 14685–14695. 38773944 10.1021/acsnano.4c03290 PMC11155241

[15] Y. Du , B. Xu , G. Wang , et al., “Review of Highly Mismatched III‐V Heteroepitaxy Growth on (001) Silicon,” Nanomaterials 12 (2022): 741. 35269230 10.3390/nano12050741 PMC8912022

[16] Z. Luo , Z. Zhang , F. Zhao , et al., “Advanced Polishing Methods for Atomic‐Scale Surfaces: A Review,” Materials Today Sustainability 27: 100841.

[17] L. Ye , J. Wu , X. Zhu , et al., “Optimization of Polishing Fluid Composition for Single Crystal Silicon Carbide by Ultrasonic Assisted Chemical‐Mechanical Polishing,” Scientific Reports 14 (2024): 26056. 39472699 10.1038/s41598-024-77598-x PMC11522676

[18] K. Yang , N. Huang , H. Di , and P. Zhou , “Modeling of Surface Microtopography Evolution in Chemical Mechanical Polishing Considering Chemical‐Mechanical Synergy,” Tribology International 201 (2025): 110206.

[19] X. Zhu , J. Ding , Z. Mo , et al., “Evaluation of Chemical Mechanical Polishing Characteristics Using Mixed Abrasive Slurry: A Study on Polishing Behavior and Material Removal Mechanism,” Applied Surface Science 679 (2025): 161157.

[20] M. Muthuvel and J. Stickney , “Surface Chemistry of InP(100) After Wet and Electrochemical Etching,” Journal of The Electrochemical Society 153 (2006): C67.

[21] J. B. Matovu , P. Ong , L. H. A. Leunissen , S. Krishnan , and S. V. Babu , “Use of Multifunctional Carboxylic Acids and Hydrogen Peroxide To Improve Surface Quality and Minimize Phosphine Evolution During Chemical Mechanical Polishing of Indium Phosphide Surfaces,” Industrial & Engineering Chemistry Research 52 (2013): 10664–10672.

[22] S. Hayashi , M. Joshi , and M. Goorsky , “Chemical Mechanical Polishing of Exfoliated III‐V Layers,” ECS Transactions 16 (2008): 295–302.

[23] J. Geng , J. Deng , X. Wen , et al., “Fundamental Study on the Chemical Mechanical Polishing for Indium Phosphide Wafers: Thermochemical Analysis, Oxidation and Chemical Mechanical Polishing Experiments,” Materials Science in Semiconductor Processing 203 (2026): 110252.

[24] M. Qi , M. Sun , and X. Yang , “Enhancement of the Effect of EDTA and GLY on InP Alkaline CMP: Removal Rate, Surface Morphology, and Theoretical Studies,” Materials Science and Engineering: B 318 (2025): 118288.

[25] S. Peddeti , P. Ong , L. H. A. Leunissen , and S. V. Babu , “Chemical Mechanical Polishing of InP,” ECS Journal of Solid State Science and Technology 1 (2012): P184–P189.

[26] R. K. Bhonsle , L. Teugels , S. A. U. Ibrahim , et al., “Inspection, Characterization and Classification of Defects for Improved CMP of III‐V Materials,” ECS Journal of Solid State Science and Technology 4 (2015): P5073–P5077.

[27] O. Bienek , B. Fuchs , M. Kuhl , et al., “Engineering Defects and Interfaces of Atomic Layer‐Deposited TiO x ‐Protective Coatings for Efficient III–V Semiconductor Photocathodes,” ACS Photonics 10 (2023): 3985–3997.

[28] S. Tian , Z. Wei , Y. Li , et al., “Surface State and Optical Property of Sulfur Passivated InP,” Materials Science in Semiconductor Processing 17 (2014): 33–37.

[29] D. Cuypers , S. De Gendt , S. Arnauts , K. Paulussen , and D. H. Van Dorp , “Wet Chemical Etching of InP for Cleaning Applications,” ECS Journal of Solid State Science and Technology 2 (2013): P185–P189.

[30] M. V. Lebedev , Y. M. Serov , T. V. Lvova , R. Endo , T. Masuda , and I. V. Sedova , “InP(100) Surface Passivation With Aqueous Sodium Sulfide Solution,” Applied Surface Science 533 (2020): 147484.

[31] Z. Wang , P. Liu , J. Lee , et al., “Investigation of Cleaning Mechanisms for Particle, Metal Ion, and Organic Contaminations in Amorphous Carbon Post‐CMP Cleaning,” Applied Surface Science 717 (2026): 164803.

[32] W. Wang , B. Zhang , Y. Shi , et al., “Improvement in Chemical Mechanical Polishing of 4H‐SiC Wafer by Activating Persulfate Through the Synergistic Effect of UV and TiO_2_ ,” Journal of Materials Processing Technology 295 (2021): 117150.

[33] Z. Xu , X. Li , J. Li , et al., “Regulation of Reaction Pathways in Coordinated Chains by Directional Mechanical Force,” ACS Nano 19 (2025): 6120–6129. 39908530 10.1021/acsnano.4c13622

[34] J. Li , S. Ju , Y. Tang , et al., “Remarkable Bias‐Stress Stability of Ultrathin Atomic‐Layer‐Deposited Indium Oxide Thin‐Film Transistors Enabled by Plasma Fluorination,” Advanced Functional Materials 34 (2024): 2401170.

[35] T. A. Colleran , A. I. Abdulagatov , J. L. Partridge , A. S. Cavanagh , and S. M. George , “Thermal Atomic Layer Etching of Indium Gallium Zinc Oxide (IGZO), In_2_O_3_, Ga_2_O3, and ZnO Using Sequential Hydrogen Fluoride and Acetylacetone Exposures,” The Journal of Physical Chemistry C 129 (2025): 20223–20233.

[36] M. Qiang , M. Wang , L. Zhang , et al., “Research on Ultra‐Smooth Surface Processing of Mid‐Infrared Indium Fluoride‐Based Fiber Glass,” Journal of Alloys and Compounds 1027 (2025): 180590.

[37] R. L. Davidovich , P. P. Fedorov , and A. I. Popov , “Structural Chemistry of Anionic Fluoride and Mixed‐Ligand Fluoride Complexes of Indium(III),” Reviews in Inorganic Chemistry 36, no. 3 (2016): 105–133.

[38] Q. Wang , L. Jiang , Y. Yu , and J. Sun , “Progress of Enhancing the Safety of Lithium Ion Battery from the Electrolyte Aspect,” Nano Energy 55 (2019): 93–114.

[39] L. Zhang , Y. Jiang , Z. Wang , et al., “Performance and Structure Evolution of Fluoindinate Glass at High Temperatures,” Journal of the American Ceramic Society 105 (2022): 2001–2009.

[40] Z. Piao , P. Xiao , R. Luo , et al., “Constructing a Stable Interface Layer by Tailoring Solvation Chemistry in Carbonate Electrolytes for High‐Performance Lithium‐Metal Batteries,” Advanced Materials 34 (2022): 2108400. 10.1002/adma.202108400 34859925

[41] Y. Qiu , P. Bellina , L. P. H. Jeurgens , et al., “Aqueous Deposition of Ultraviolet Luminescent Columnar Tin‐Doped Indium Hydroxide Films,” Advanced Functional Materials 18 (2008): 2572–2583.

[42] R. Wu , X. Bo , S. Zhao , et al., “Transition Metal Fluorides as Advanced Cathodes for Lithium/Sodium‐Ion Batteries: Rational Enhancement Strategies and Underlying Electrochemical Mechanisms,” Advanced Functional Materials 35 (2025): 2424603.

[43] F. S. Tschernuth , T. Thorwart , L. Greb , F. Hanusch , and S. Inoue , “Bis(perfluoropinacolato)silane: A Neutral Silane Lewis Superacid Activates Si−F Bonds,” Angewandte Chemie International Edition 60 (2021): 25799–25803. 34570964 10.1002/anie.202110980 PMC9298387

[44] Y. Chen , Y. Gao , H. Chen , et al., “Engineering Inorganic Nanoemulsions/Nanoliposomes by Fluoride‐Silica Chemistry for Efficient Delivery/Co‐Delivery of Hydrophobic Agents,” Advanced Functional Materials 22 (2012): 1586–1597.

[45] X. Zhang , N. Meng , X. Li , X. Mei , L. Yang , and Y. He , “The Role of Ammonium Citrate and Dodecyl Pyridinium Chloride on Chemical Mechanical Polishing Relevant to SiO_2_ Dielectric Layer,” Journal of Manufacturing Processes 107 (2023): 333–344.

[46] M. Lo , T. Ng , H. Mo , and C. Lee , “Direct Threat of a UV‐Ozone Treated Indium‐Tin‐Oxide Substrate to the Stabilities of Common Organic Semiconductors,” Advanced Functional Materials 23 (2013): 1718–1723.

[47] P. Liu , Y. Lou , S. Ding , et al., “Green InP/ZnSeS/ZnS Core Multi‐Shelled Quantum Dots Synthesized with Aminophosphine for Effective Display Applications,” Advanced Functional Materials 31 (2021): 2008453.

[48] J. Deng , Z. Zhang , Y. Bian , et al., “Dual‐Surface Treatment for Efficient Amine‐Phosphine Based Red InP QLEDs,” Advanced Functional Materials 36 (2025): 21226.

[49] Z. Sun , Q. Hou , J. Kong , et al., “Surface Passivation toward Multiple Inherent Dangling Bonds in Indium Phosphide Quantum Dots,” Inorganic Chemistry 63 (2024): 6396–6407. 38528328 10.1021/acs.inorgchem.4c00168

[50] V. Hrubý , L. Zdražil , J. Dzíbelová , et al., “Unveiling the True Band Gap of Fluorographene and its Origins by Teaming Theory and Experiment,” Applied Surface Science 587 (2022): 152839.

[51] S. Sadeghi , H. Bahmani Jalali , R. Melikov , et al., “Stokes‐Shift‐Engineered Indium Phosphide Quantum Dots for Efficient Luminescent Solar Concentrators,” ACS Applied Materials & Interfaces 10 (2018): 12975–12982. 29589740 10.1021/acsami.7b19144 PMC5997383

[52] E. M. Janke , N. E. Williams , C. She , et al., “Flipping the Switch: Fast Photoisomerization in a Confined Environment,” Journal of the American Chemical Society 140 (2018): 7611–7622. 29807417 10.1021/jacs.8b02994

[53] Z. He , J. Zhou , Y. Qi , C. Luo , C. Wang , and J. Liu , “Triple Synergism Effect of Ammonium Nitrilotriacetate on the Chemical Mechanical Polishing Performance of Ruthenium Barrier Layers,” Small 20 (2024): 2309965. 10.1002/smll.202309965 38247206

[54] S. Wang , J. Lan , Z. Ren , et al., “Molecular Dynamics Simulations of Hybrid Cell Membrane‐Coated Porphyrin Nanoparticles for Enhanced Photochemotherapy of Breast Cancer,” Advanced Functional Materials 35 (2025): 2425101.

[55] Y. He , M. Wang , H. Ji , et al., “Molecular Dynamics Simulations for Electrocatalytic CO_2_ Reduction: Bridging Macroscopic Experimental Observations and Microscopic Explanatory Mechanisms,” Advanced Functional Materials 35 (2025): 2413703.

[56] T. Zhong , T. Xu , L. Zhang , L. Wang , F. Wu , and X. Yu , “Modulation on Surface Termination Groups to Optimize the Adsorption Energy and Work Function of Nb_2_CT_x_ for Enhanced Hydrogen Storage in Magnesium Hydride,” Advanced Functional Materials 35 (2025): 2418230.

