# Supplementary material for: One‐Step, High‐Removal‐Rate and Low‐Damage Chemical Mechanical Polishing of InP Enabled by Hydrolysis Activated PF6 − with In Situ Fluoride Passivation
Source: Adv Sci (Weinh). 2026 Apr 2;13(36):e75142. doi: 10.1002/advs.75142 (PMC13317576; doi:10.1002/advs.75142)
Supplement: Supplementary file 1 — Supporting File: advs75142‐sup‐0001‐SuppMat.docx. [file ADVS-13-e75142-s001.docx]

**Supporting Information**

**One-Step, High-Removal-Rate and Low-Damage Chemical Mechanical Polishing of InP Enabled by Hydrolysis Activated PF₆⁻ with In Situ Fluoride Passivation**

*Shigong Fu^12^, Jianwei Zhou^12^, Chenwei Wang^12^, Chong Luo^12^, Yuhang Qi^12*^*

所，上海，201204(中国)中国科学院上海高级研究院上海同步辐射装置，上海，

^1^School of Electronic Information Engineering, Hebei University of Technology, Tianjin 300130, China

^2^Tianjin Key Laboratory of Electronic Materials and Devices, Tianjin 300130, China

*Corresponding author email: yuhangqi@hebut.edu.cn

**Supplementary Figures**

[Fig. S1. Dependence of the InP MRR and Sq on CMP parameters: (a) SiO₂ particle diameter, (b) SiO₂ abrasive concentration, (c) downforce pressure, (d) polishing-head rotation speed, (e) platen rotation speed, (f) polishing time. 4](#_Toc223691724)

[Fig. S2. AFM images of InP surfaces polished with SiO₂ abrasives of different particle sizes. 5](#_Toc223691725)

[Fig. S3. AFM images of InP wafer surfaces polished at different silica‑sol concentrations. 6](#_Toc223691726)

[Fig. S4. AFM images of InP surfaces polished under varying downforce pressures. 7](#_Toc223691727)

[Fig. S5. AFM images of InP surfaces polished at different polishing‑head rotation speeds. 8](#_Toc223691728)

[Fig. S6. AFM images of InP surfaces polished at different platen rotation speeds. 9](#_Toc223691729)

[Fig. S7. AFM images of InP surfaces obtained after polishing for different durations. 10](#_Toc223691730)

[Fig. S8. AFM images of InP surfaces polished at different slurry flow rates. 11](#_Toc223691731)

[Fig. S9. AFM images of InP surfaces polished using varying NH₄PF₆ concentrations. 12](#_Toc223691732)

[Fig. S10. AFM images of InP surfaces polished in slurries of different pH values. 13](#_Toc223691733)

[Fig. S11. Effect of NH₄PF₆ at Different Concentrations on Sq and MRR of InP Wafers. 14](#_Toc223691734)

[Fig. S12. Effect of pH in a 1% NH_4_PF_6_ solution on (a) surface tension and (b) contact angle on InP. 15](#_Toc223691735)

[Fig. S13. Effect of NH_4_PF_6_ concentration at pH 3 on (a) surface tension and (b) contact angle on InP. 16](#_Toc223691736)

[Fig. S14. NH_4_PF_6_ was dissolved in deuterium oxide, and In(NO_3_)_3_ samples were immersed in the solution for 5 min before nuclear magnetic resonance (NMR) measurements. Shown are: (a) ^31^P spectrum, (b) ^19^F spectrum, and (c) ^1^H spectrum. 17](#_Toc223691737)

[Fig. S15. Potential-pH diagram for the In-H₂O system. 18](#_Toc223691738)

[Fig.S16. TEM images of SiO₂ (a) before and (b) after immersion in NH_4_PF_6_. Abrasive boundary trend maps extracted by Fourier transform (a) before and (b) after immersion in NH_4_PF_6_. 19](#_Toc223691739)

[Fig. S17. Effect of pH (a) and NH_4_PF_6_ (b) concentration on the silica sol particle size in the polishing slurry. 20](#_Toc223691740)

[Fig. S18. (a) Polishing performance as a function of time. (b) Slurry particle size as a function of time. 21](#_Toc223691741)

[Fig. S19. AFM of the polishing slurry over a one-week period. 22](#_Toc223691742)

[Fig. S20. Contact angle of aqueous solution on InP surface before and after polishing. 23](#_Toc223691743)

[Fig. S21. EDS spectra of InP wafers: (a) untreated, and (b) after soaking in NH₄PF₆. 24](#_Toc223691744)

[Fig. S22. Electrochemical and surface-analysis results for InP in different reagent solutions. Electrochemical measurements: (a) OCP, (b) Tafel, (c) Bode, and (d) Nyquist plots. XPS spectra: (e) In 3d and (f) P 2p. (g) Corresponding corrosion rates. 25](#_Toc223691745)

[Fig. S23. MRR and Sq of Different Reagents. 26](#_Toc223691746)

[Fig. S24. XPS of KPF_6_, NH_4_F, NH_4_H_2_PO_4_ and NH_4_H_2_PO_4_+KF: (a) O 1s (b) N 1s (c) F 1s. 27](#_Toc223691747)

[Fig. S25. SEM images of InP surfaces after soaking treatments: (a) KPF₆, (b) NH₄F, (c) NH₄H₂PO₄, and (d) NH₄H₂PO_4_+KF. 28](#_Toc223691748)

[Fig. S26. Computational analysis of interactions between selected ions/molecules and InP. (a) Optimized structures and frontier-orbital (HOMO/LUMO) density distributions. (b) Electrostatic potential maps and Fukui dual descriptors. (c) Adsorption configurations on In-terminated and P-terminated InP surfaces. 29](#_Toc223691749)

[Fig. S27. UV–vis integrating sphere absorption spectra of InP wafers before and after CMP polishing with NH₄PF₆. 30](#_Toc223691750)

[Fig. S28. UV–vis integrating‑sphere reflection spectra of InP wafers before and after CMP polishing with NH₄PF₆. 31](#_Toc223691751)

[Fig. S29. XRD patterns of InP wafers before and after CMP polishing with NH₄PF₆. 32](#_Toc223691752)

[Fig. S30. Photoluminescence excitation scans of InP wafers before and after NH₄PF₆ polishing. 33](#_Toc223691753)

[Fig. S31. PL spectra under different reagents at pH 3. 34](#_Toc223691754)

[Fig. S32. XPS analysis of InP surfaces after CMP with NH₄PF₆ and with a commercial polishing solution: (a) In 3d spectra and (b) P 2p spectra. 35](#_Toc223691755)

[Fig. S33. PLQY of commercial InP final‑polishing slurry. 36](#_Toc223691756)

[Fig. S34. TRPL of InP wafers polished with NH_4_PF_6_ and with a commercial polishing slurry. 37](#_Toc223691757)

**Supplementary Tables**

[Table. S1. The effect of different concentrations of NH4PF6 on the Ecorr and Icorr of InP. 38](#_Toc223691758)

[Table. S2. Impedance parameters of InP obtained by EEC fitting. 39](#_Toc223691759)

[Table. S3. Electrical conductivity of NH_4_PF_6_ solutions at different concentrations. 40](#_Toc223691760)

[Table. S4. Effects of different chemical reagents on the *E*_cor_r and *I*_corr_ of InP. 41](#_Toc223691761)

[Table. S5. Impedance parameters of InP obtained by EEC fitting. 42](#_Toc223691762)

[Table. S6. Peak spectral parameters of PL spectra. 43](#_Toc223691763)

**Supplementary Figures**

**
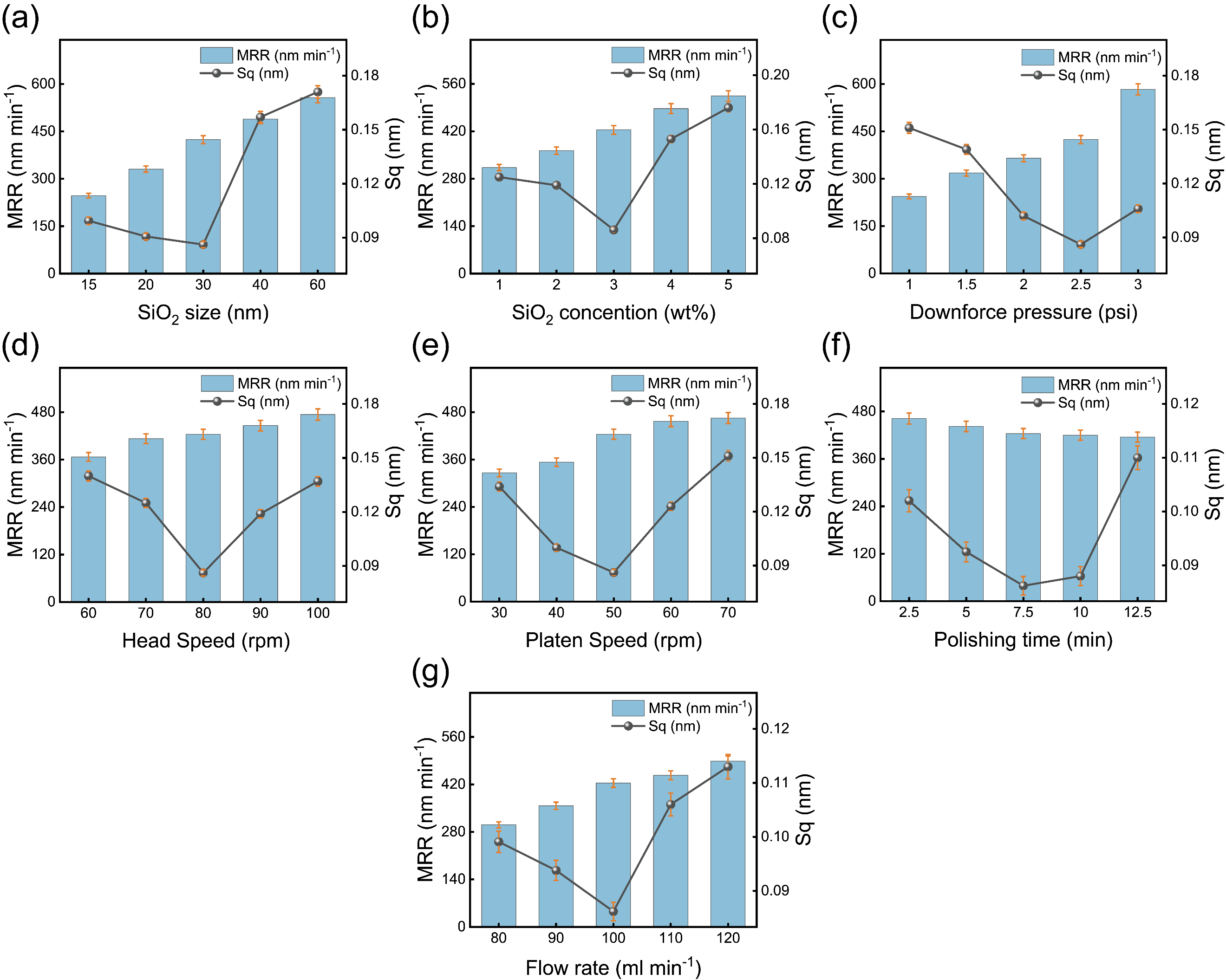
**

Fig. S1. Dependence of the InP MRR and Sq on CMP parameters: (a) SiO₂ particle diameter, (b) SiO₂ abrasive concentration, (c) downforce pressure, (d) polishing-head rotation speed, (e) platen rotation speed, (f) polishing time.


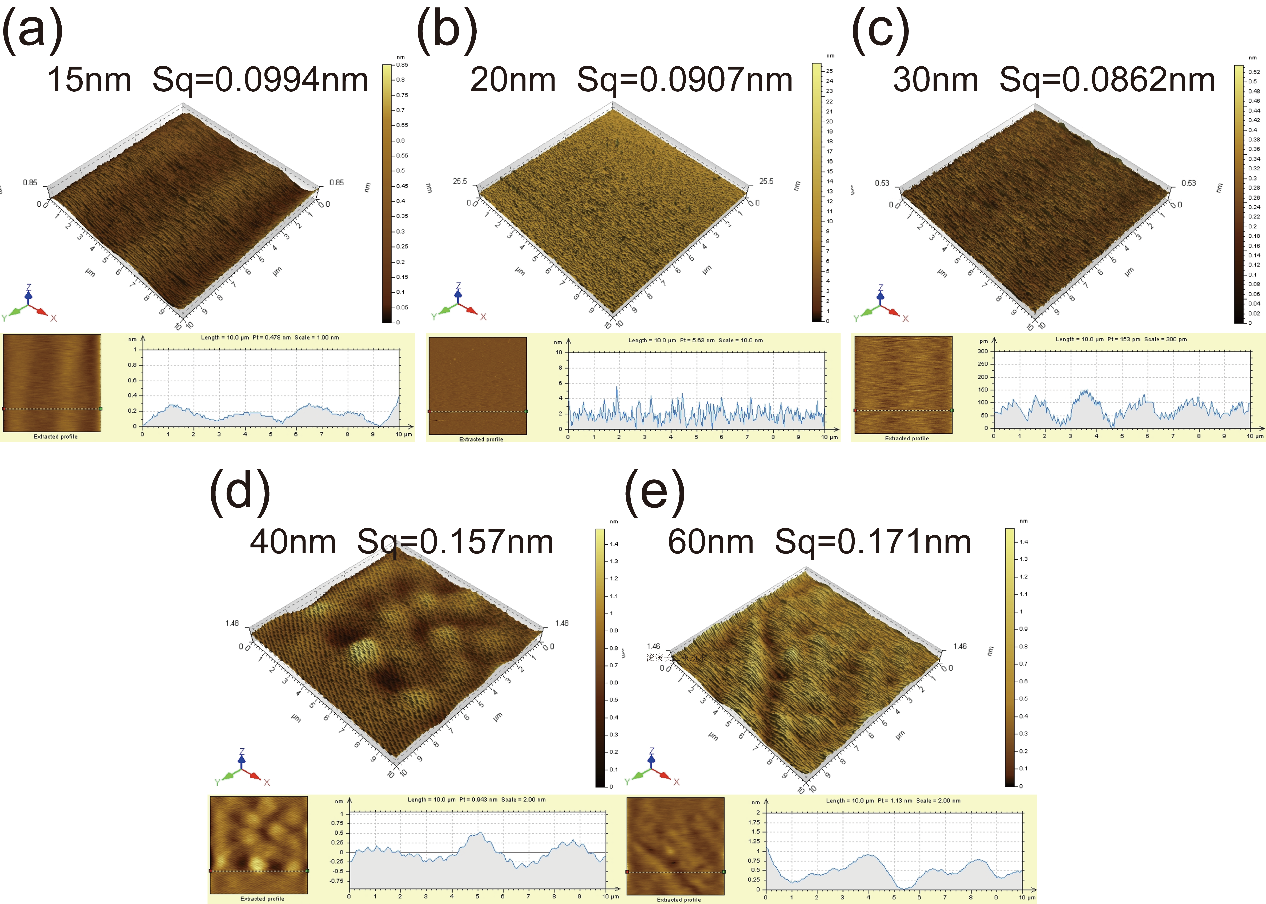


**Fig. S2.** AFM images of InP surfaces polished with SiO₂ abrasives of different particle sizes.


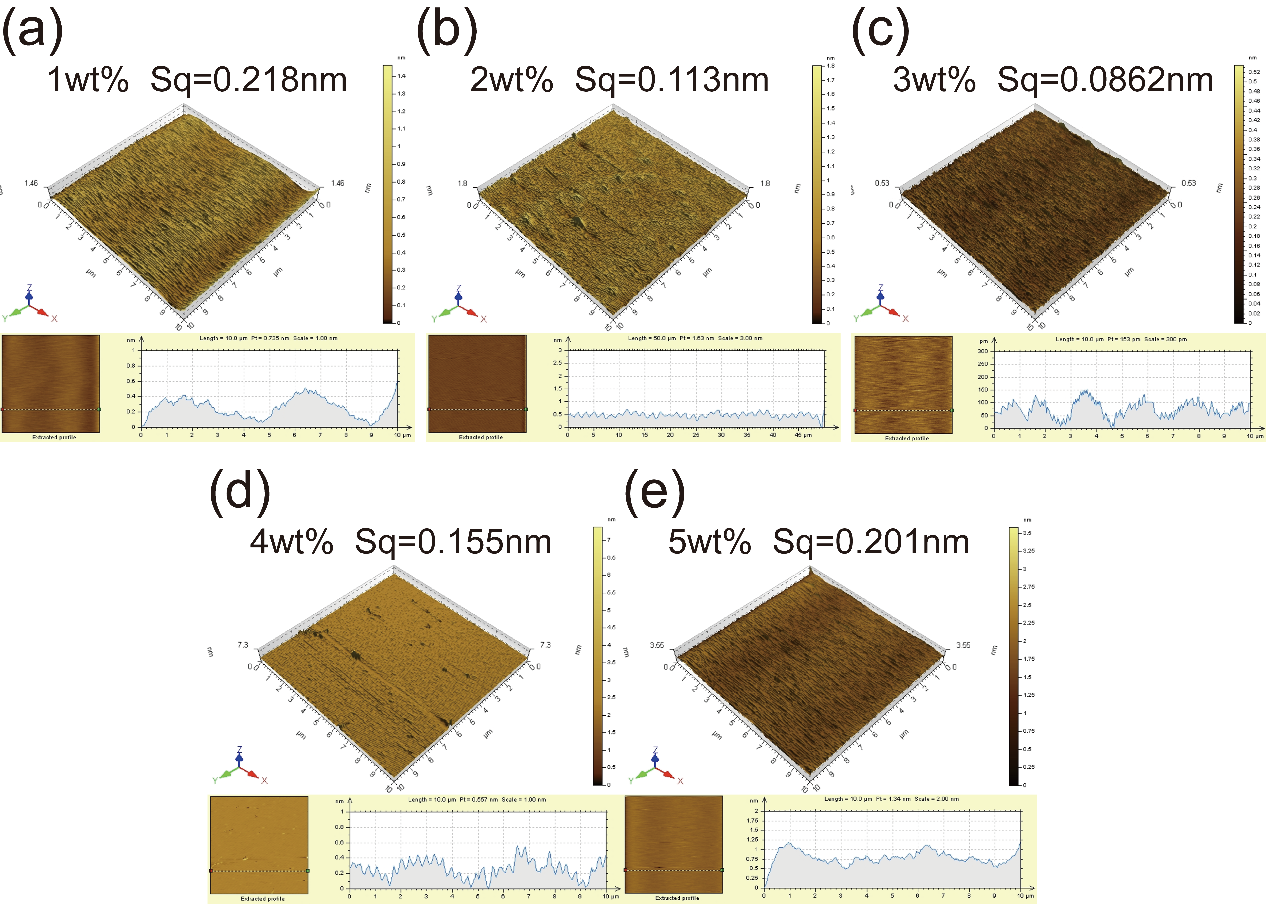


Fig. S3. AFM images of InP wafer surfaces polished at different silica‑sol concentrations.


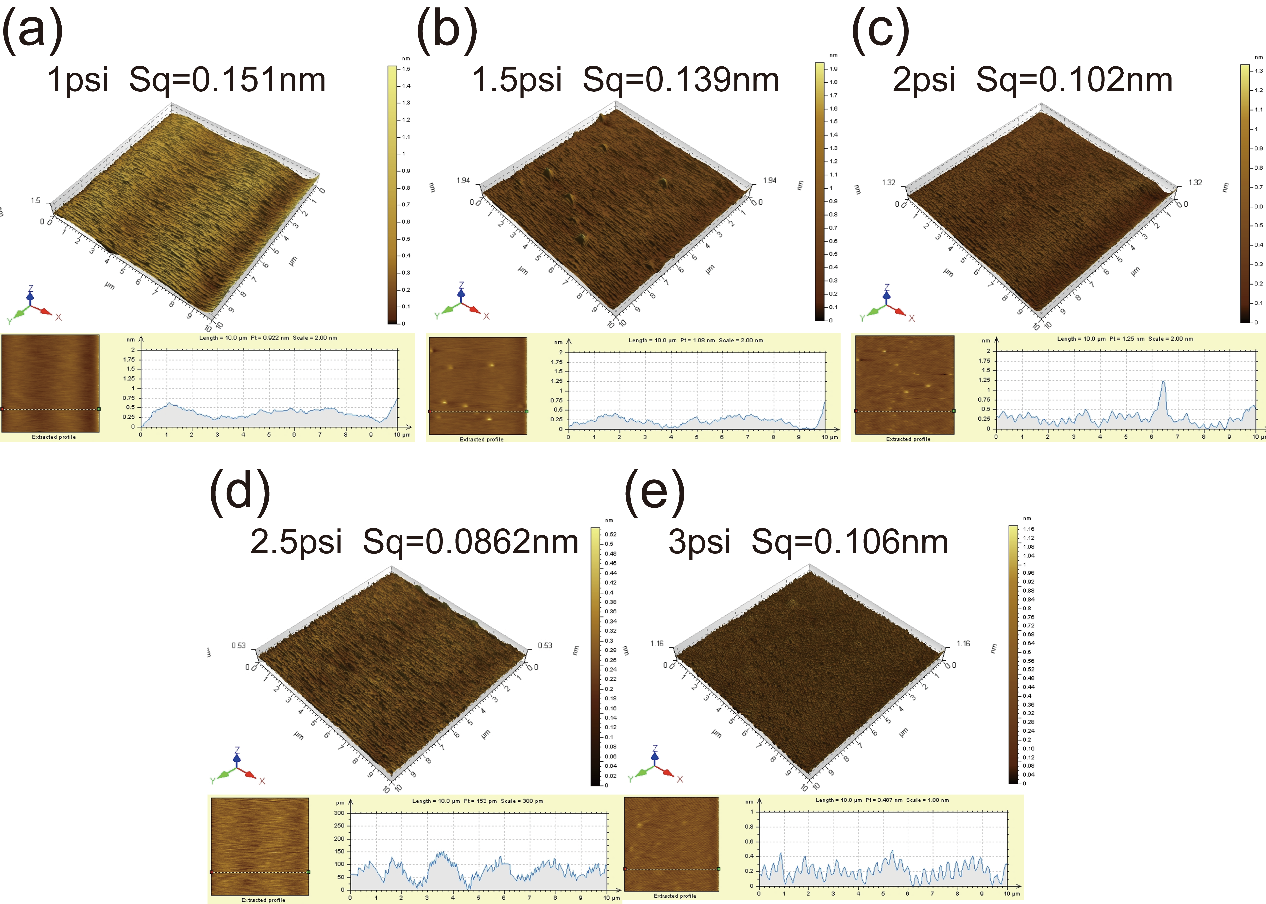


Fig. S4. AFM images of InP surfaces polished under varying downforce pressures.


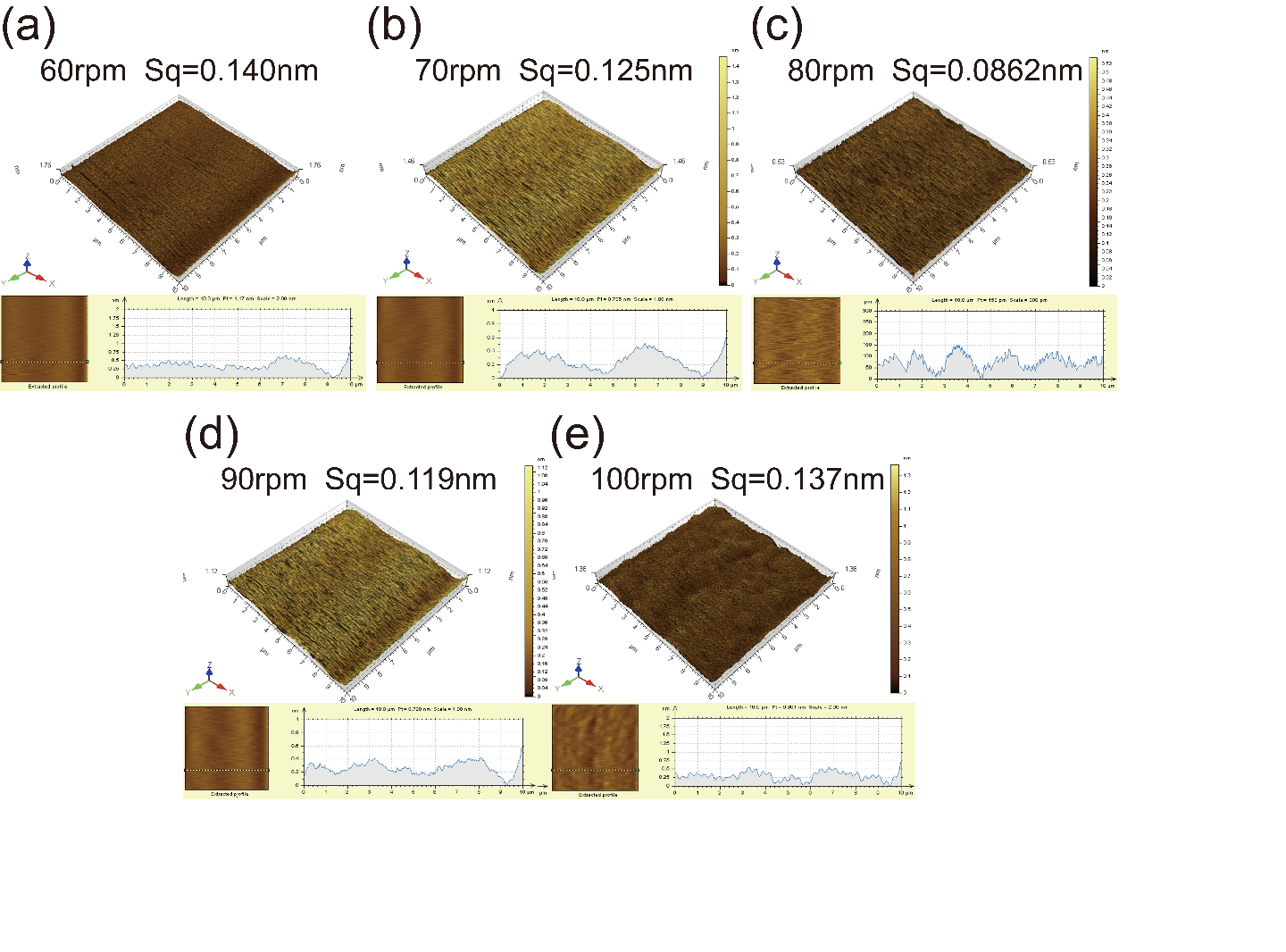


Fig. S5. AFM images of InP surfaces polished at different polishing‑head rotation speeds.


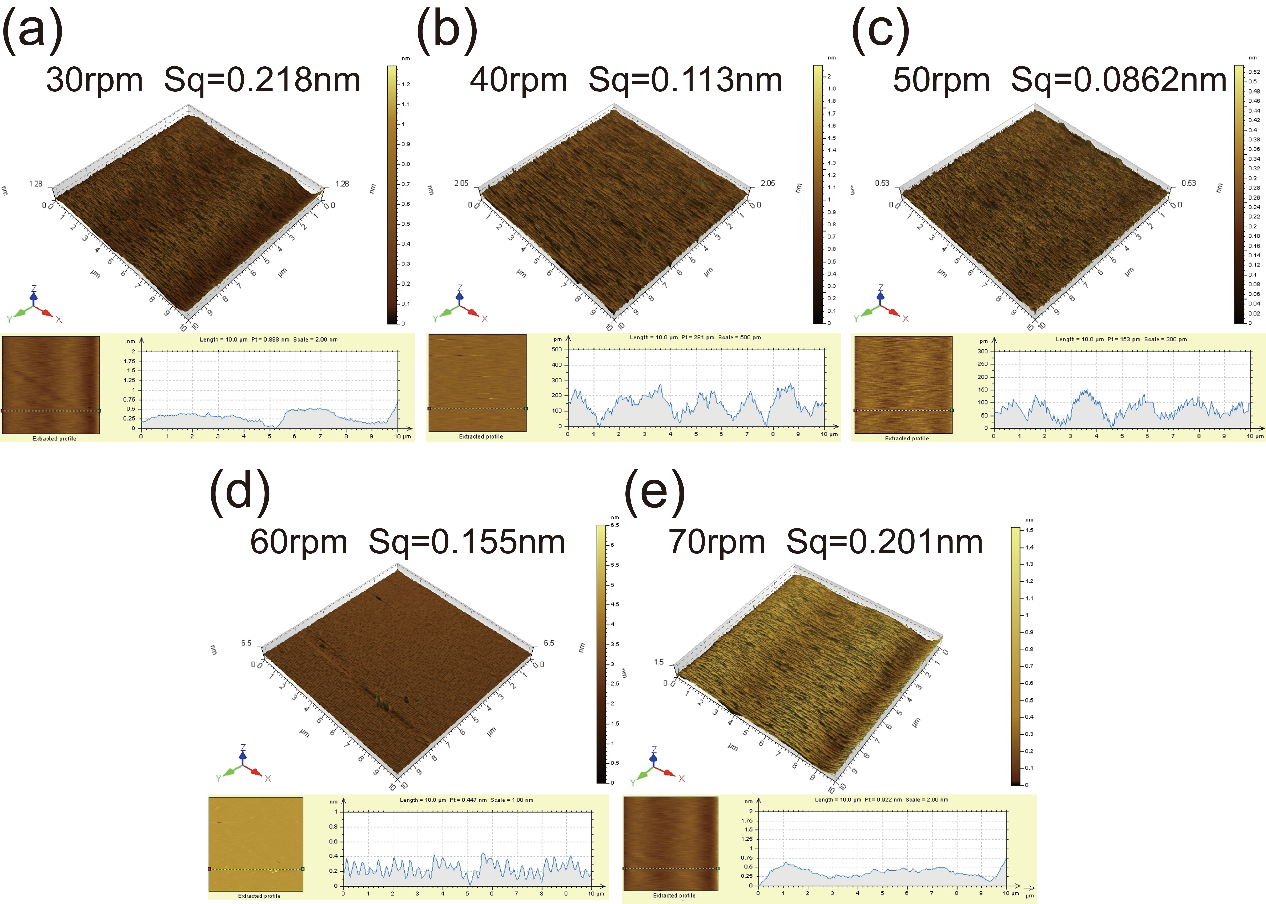


Fig. S6. AFM images of InP surfaces polished at different platen rotation speeds.


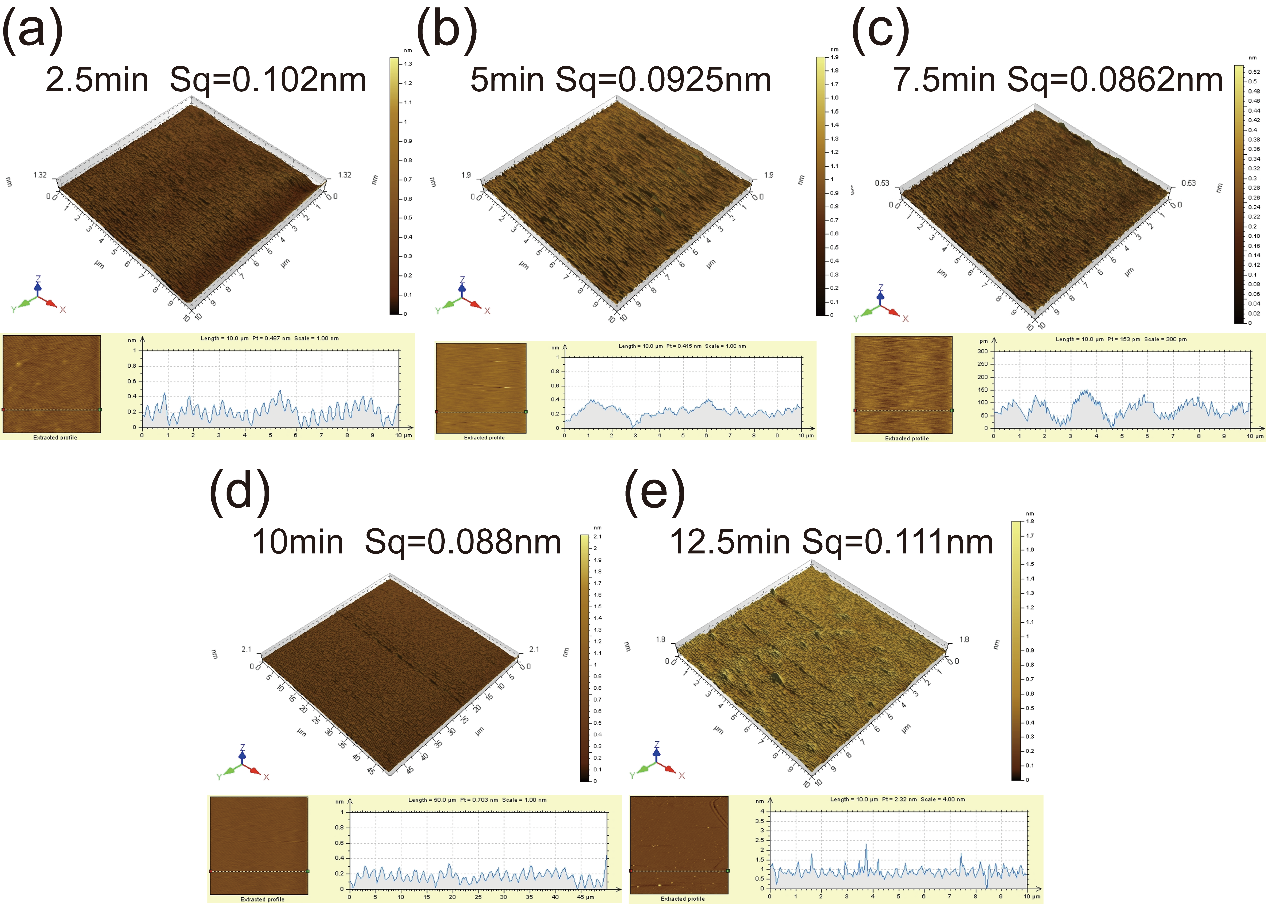


Fig. S7. AFM images of InP surfaces obtained after polishing for different durations.


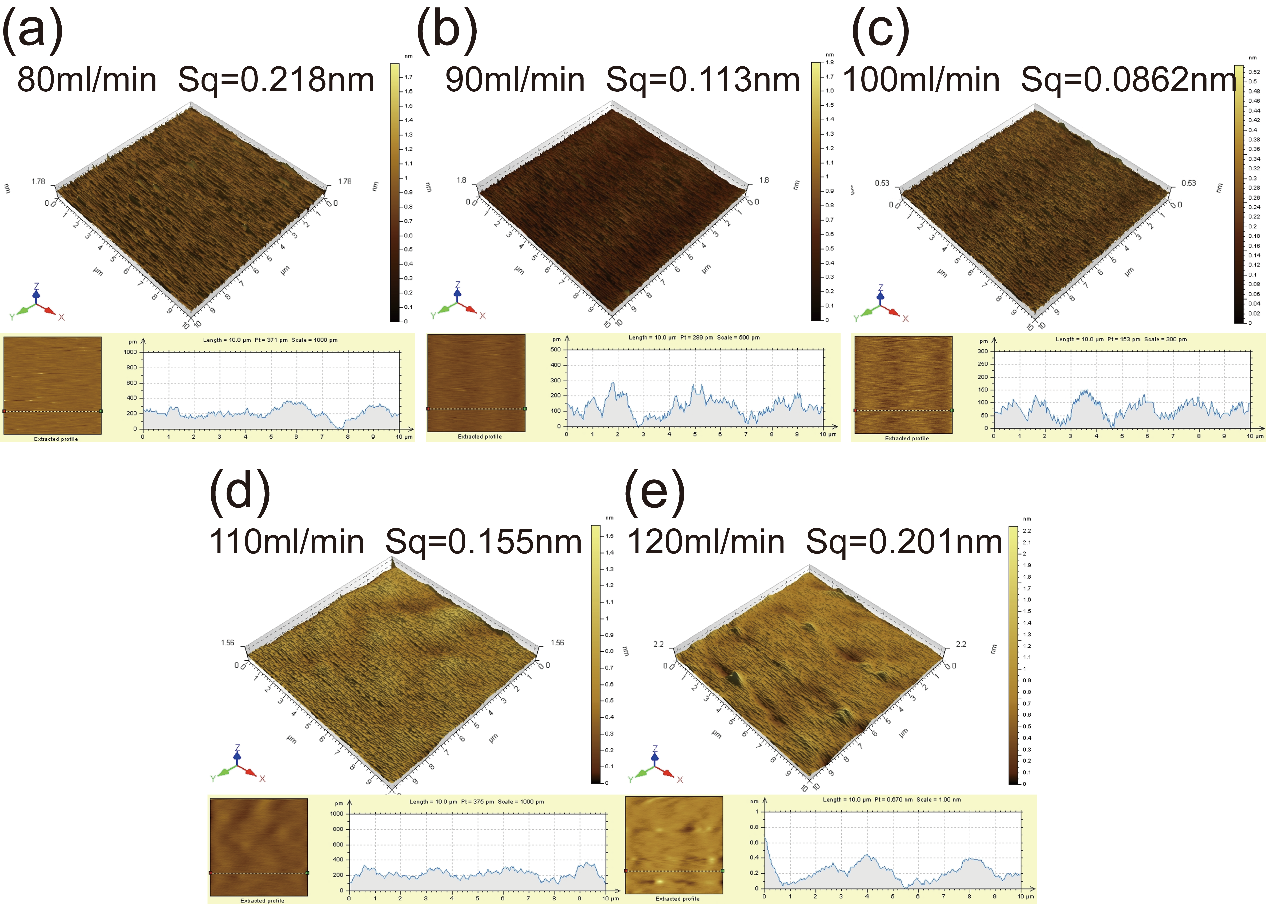


Fig. S8. AFM images of InP surfaces polished at different slurry flow rates.


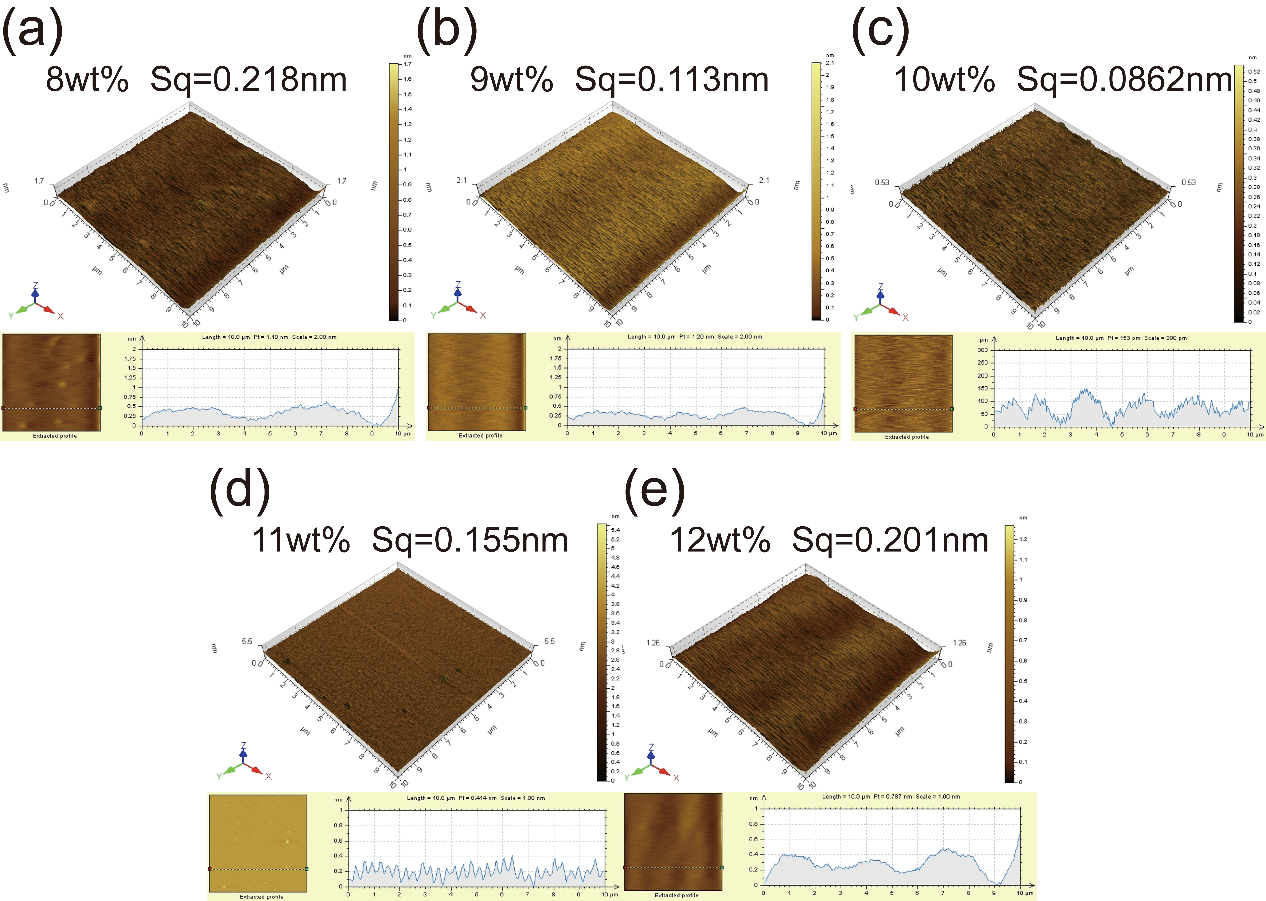


Fig. S9. AFM images of InP surfaces polished using varying NH₄PF₆ concentrations.


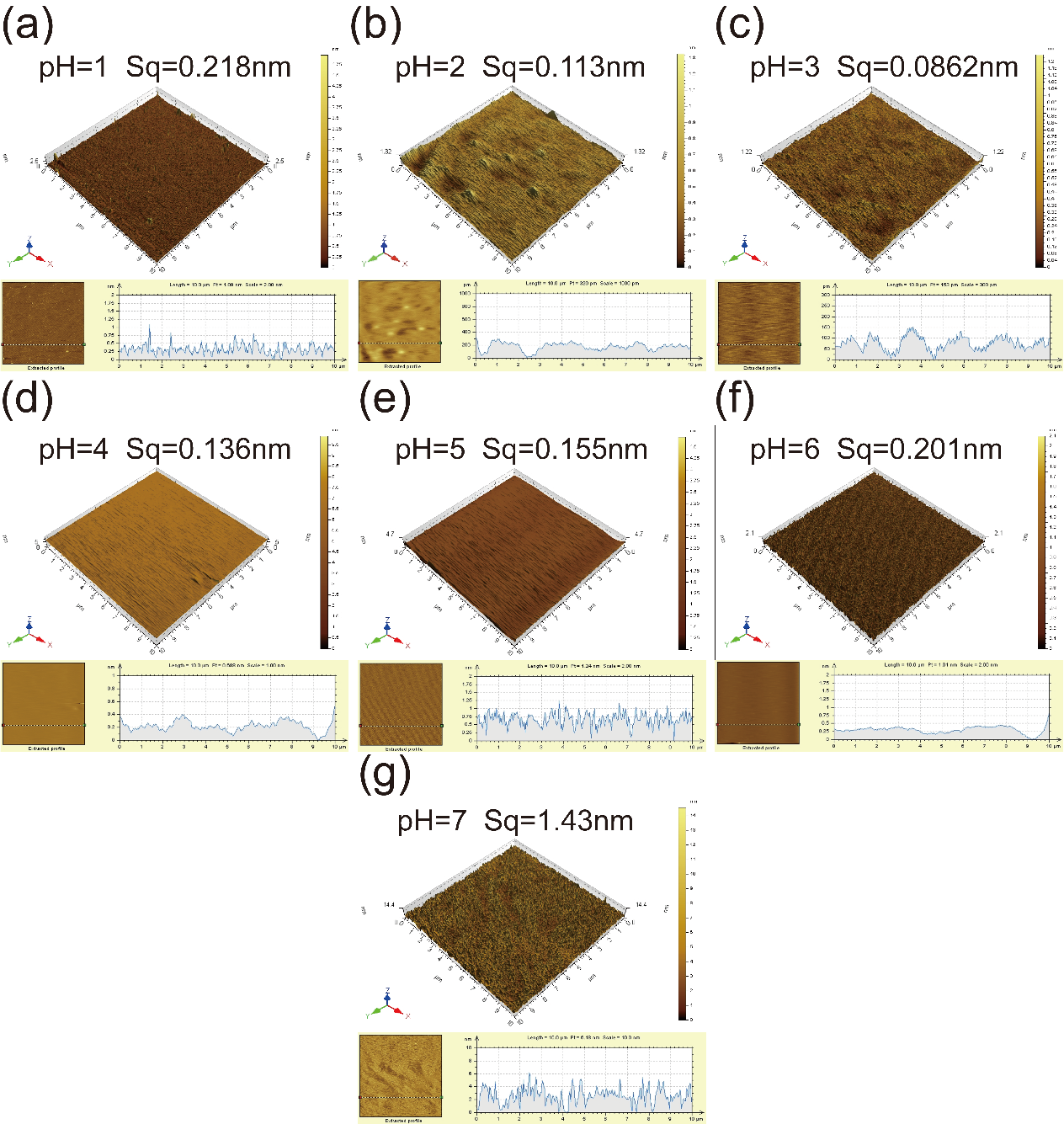


Fig. S10. AFM images of InP surfaces polished in slurries of different pH values.


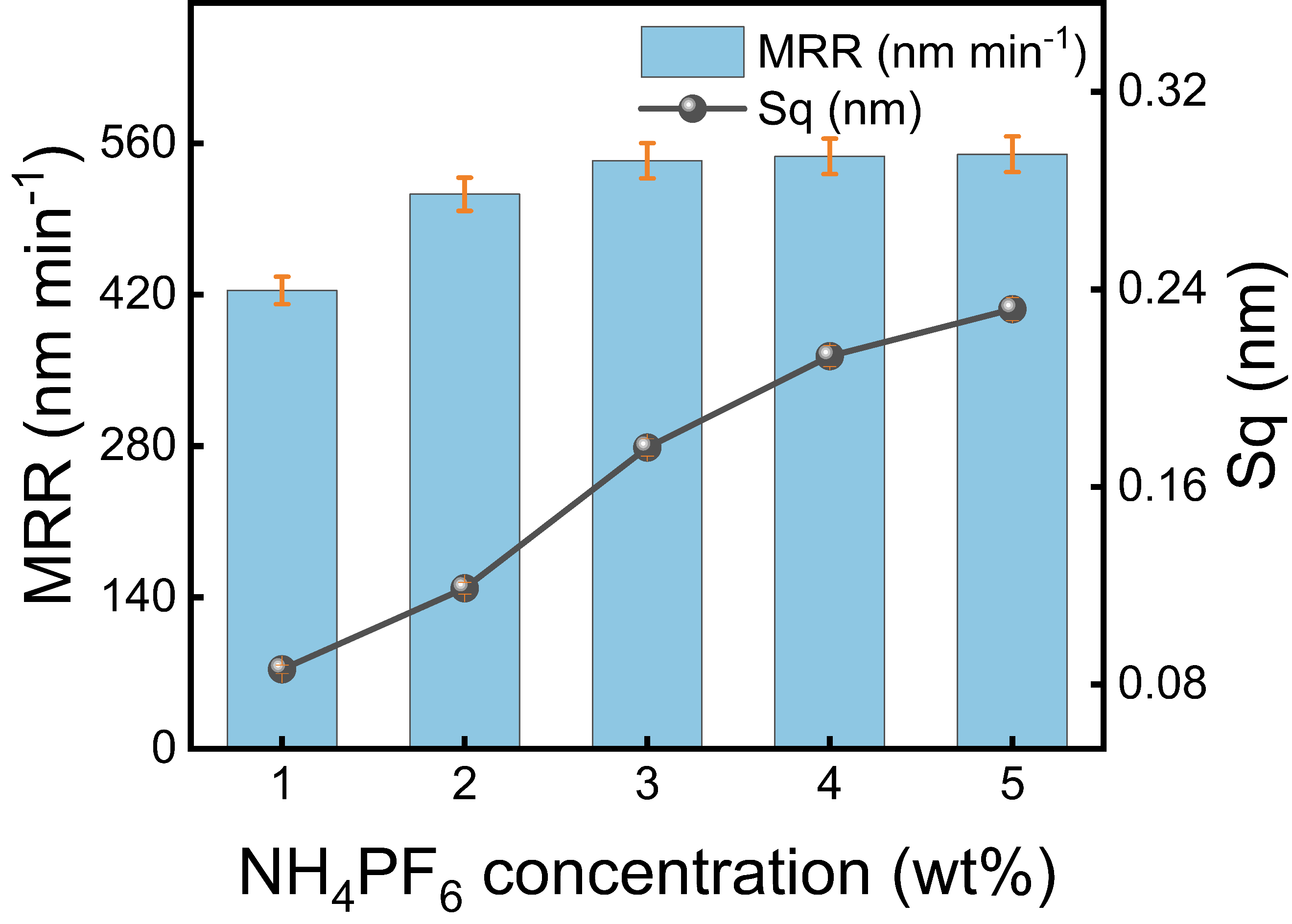


Fig. S11. Effect of NH₄PF₆ at Different Concentrations on Sq and MRR of InP Wafers.


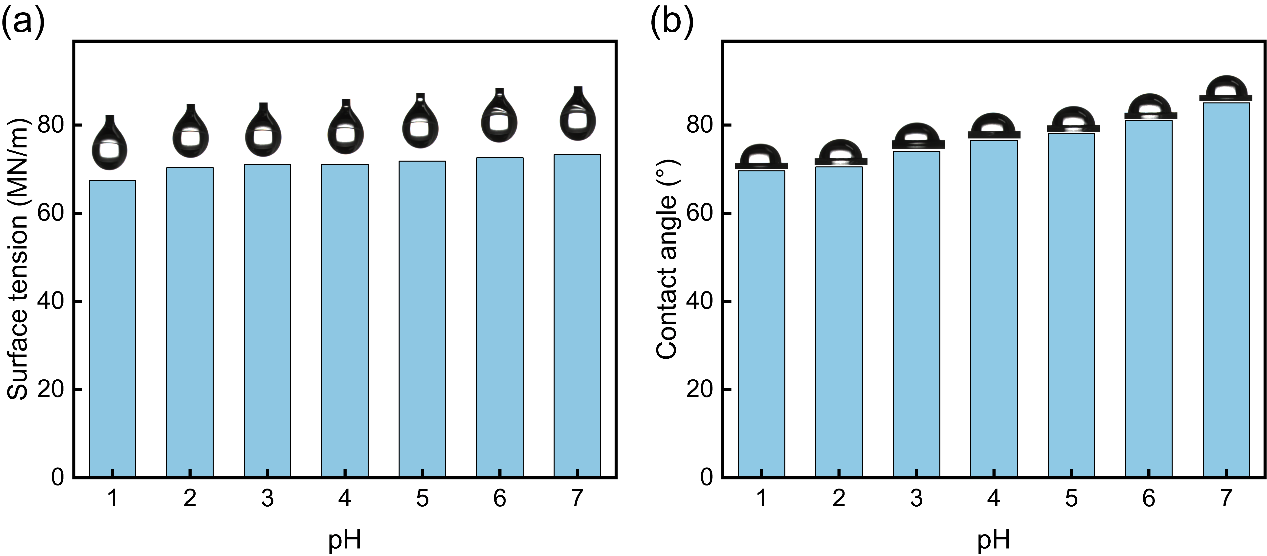


Fig. S12. Effect of pH in a 1% NH_4_PF_6_ solution on (a) surface tension and (b) contact angle on InP.


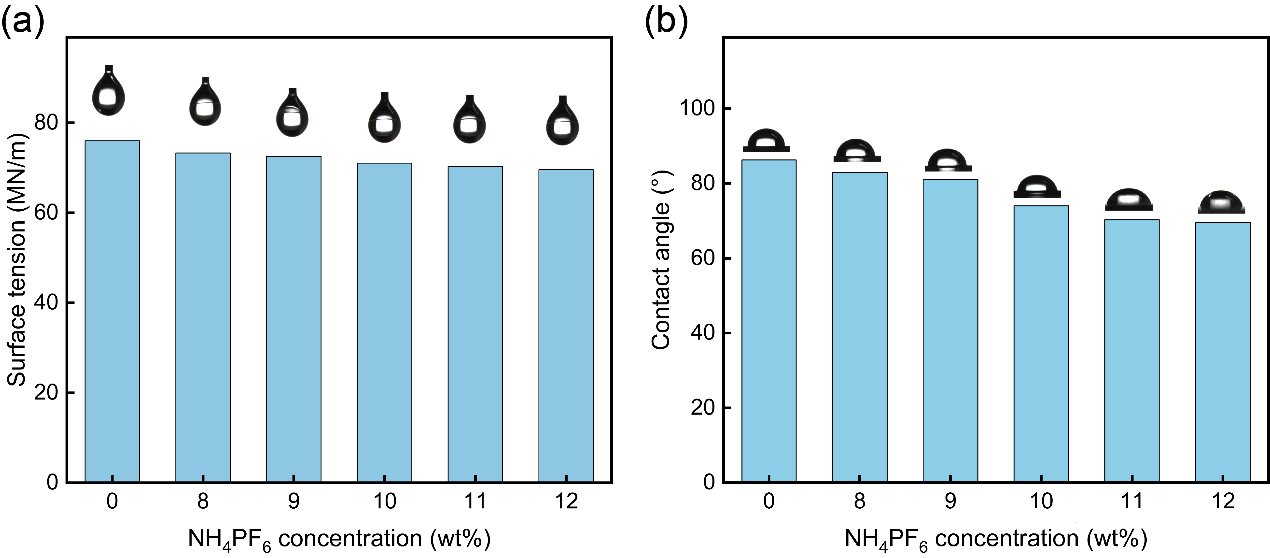


Fig. S13. Effect of NH_4_PF_6_ concentration at pH 3 on (a) surface tension and (b) contact angle on InP.


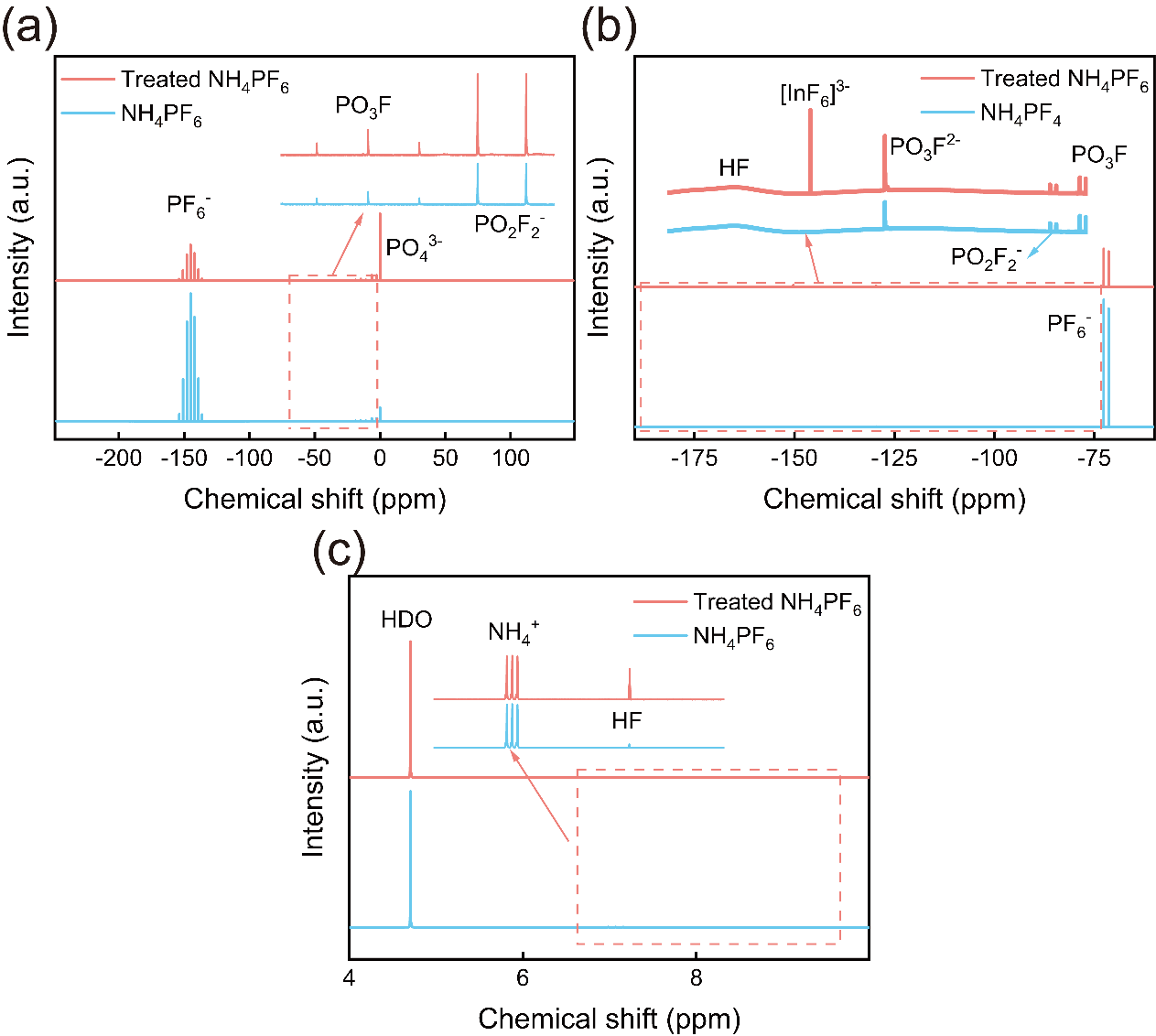


Fig. S14. NH_4_PF_6_ was dissolved in deuterium oxide, and In(NO_3_)_3_ samples were immersed in the solution for 5 min before nuclear magnetic resonance (NMR) measurements. Shown are: (a) ^31^P spectrum, (b) ^19^F spectrum, and (c) ^1^H spectrum.





Fig. S15. Potential-pH diagram for the In-H₂O system.


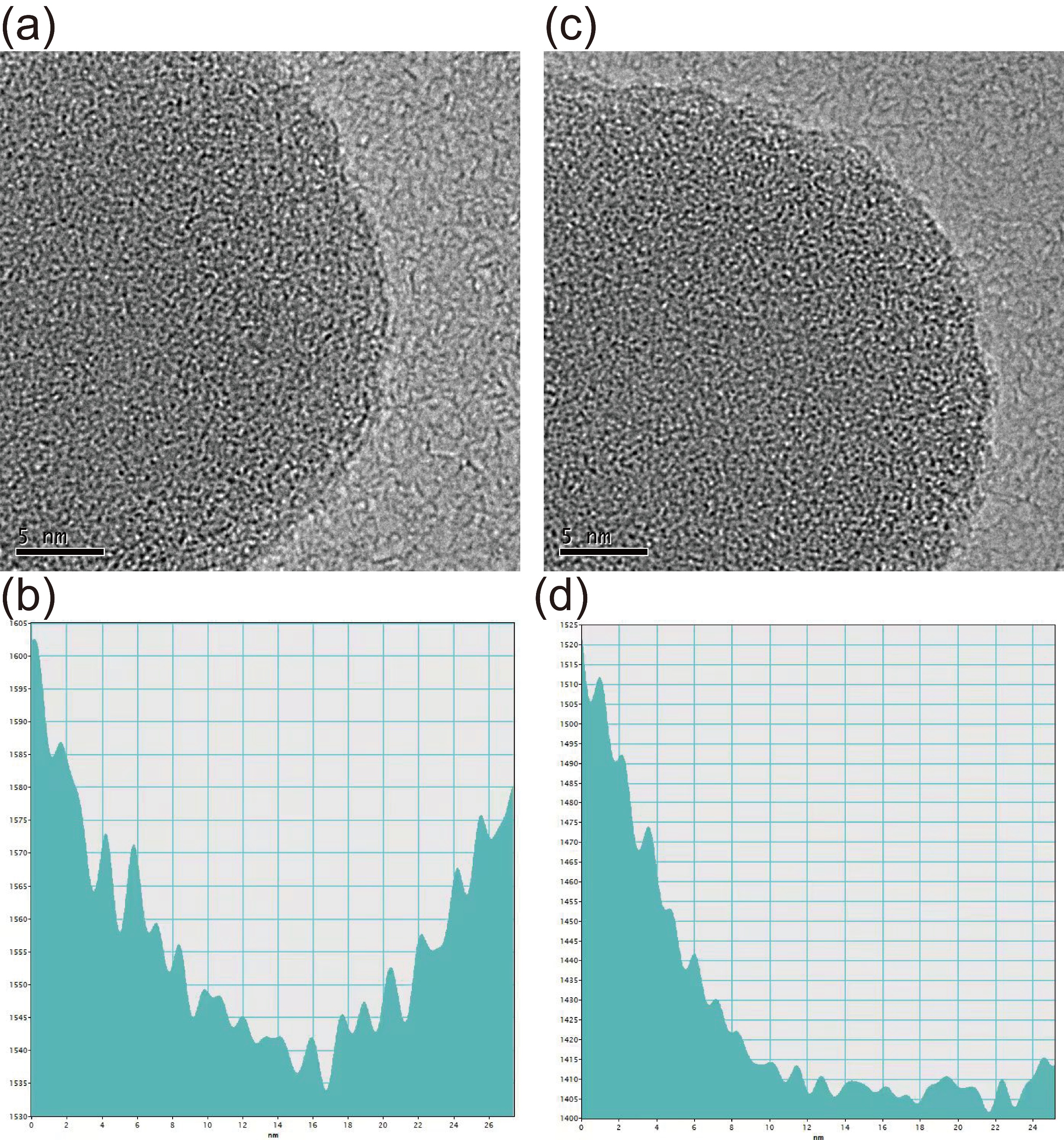


Fig.S16. TEM images of SiO₂ (a) before and (b) after immersion in NH_4_PF_6_. Abrasive boundary trend maps extracted by Fourier transform (a) before and (b) after immersion in NH_4_PF_6_.


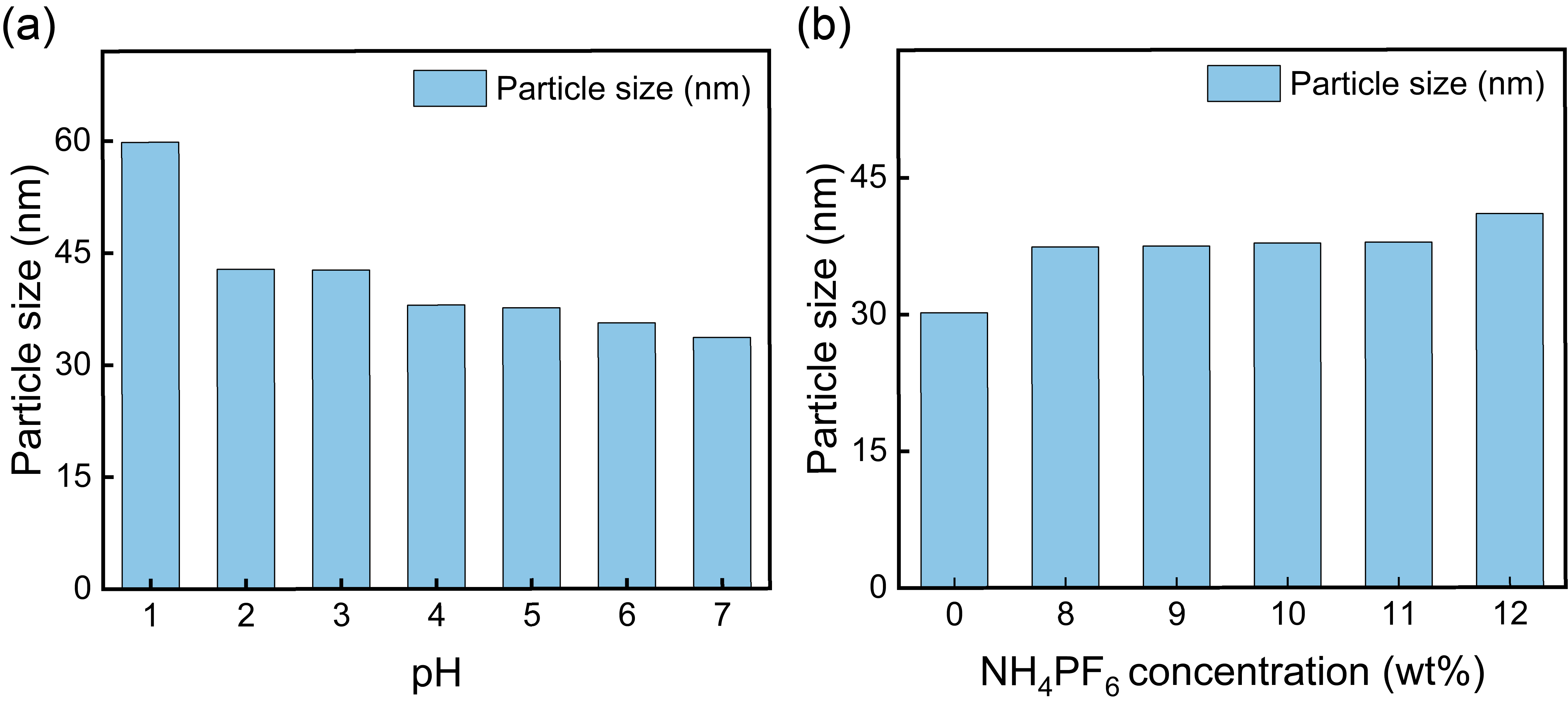


Fig. S17. Effect of pH (a) and NH_4_PF_6_ (b) concentration on the silica sol particle size in the polishing slurry.


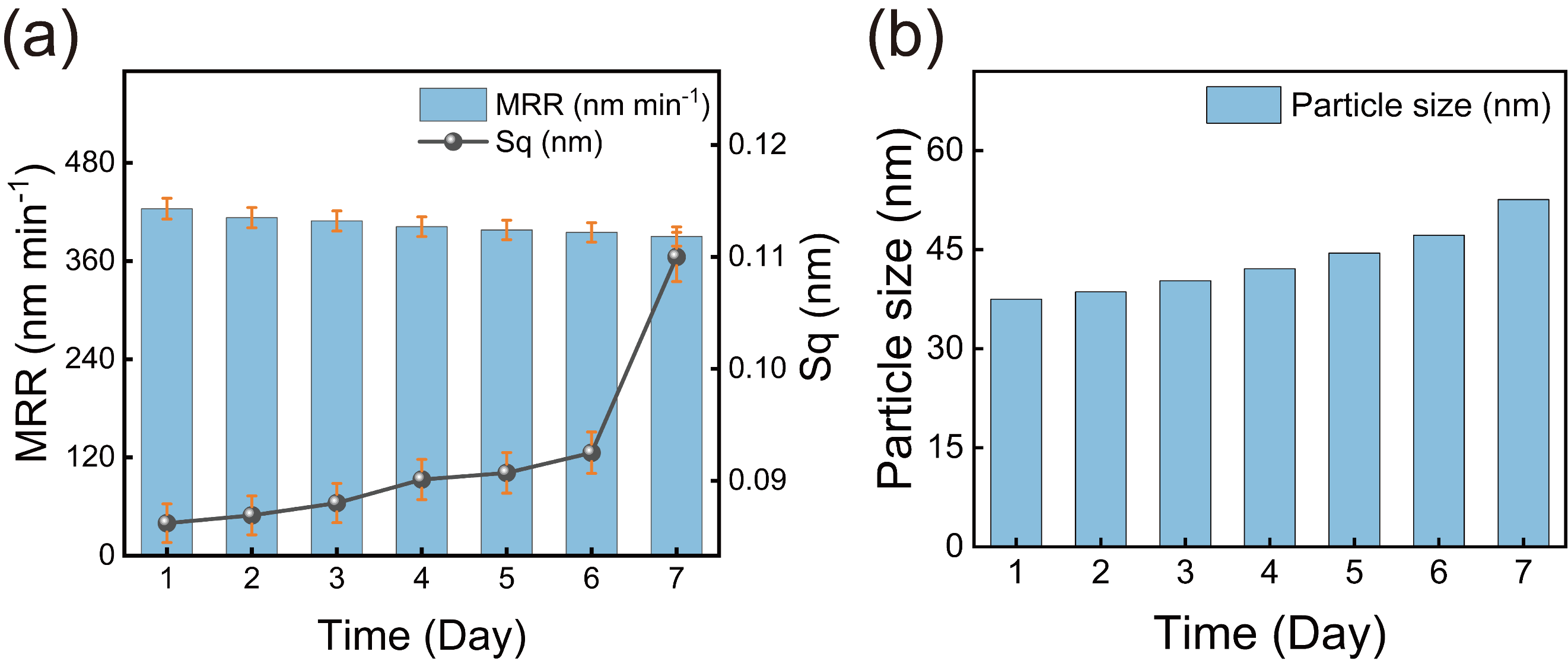


Fig. S18. (a) Polishing performance as a function of time. (b) Slurry particle size as a function of time.


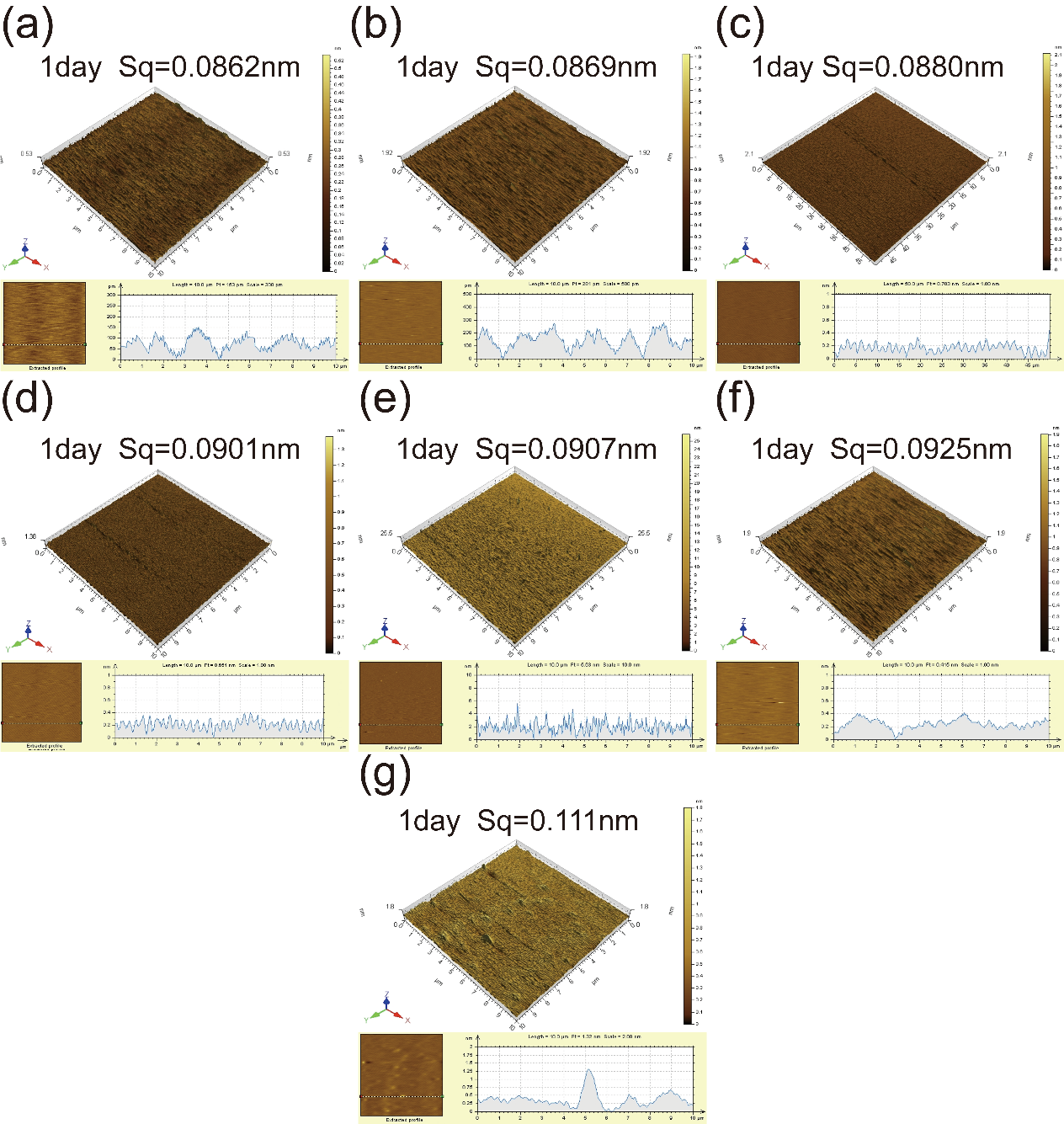


Fig. S19. AFM of the polishing slurry over a one‑week period.





Fig. S20. Contact angle of aqueous solution on InP surface before and after polishing.


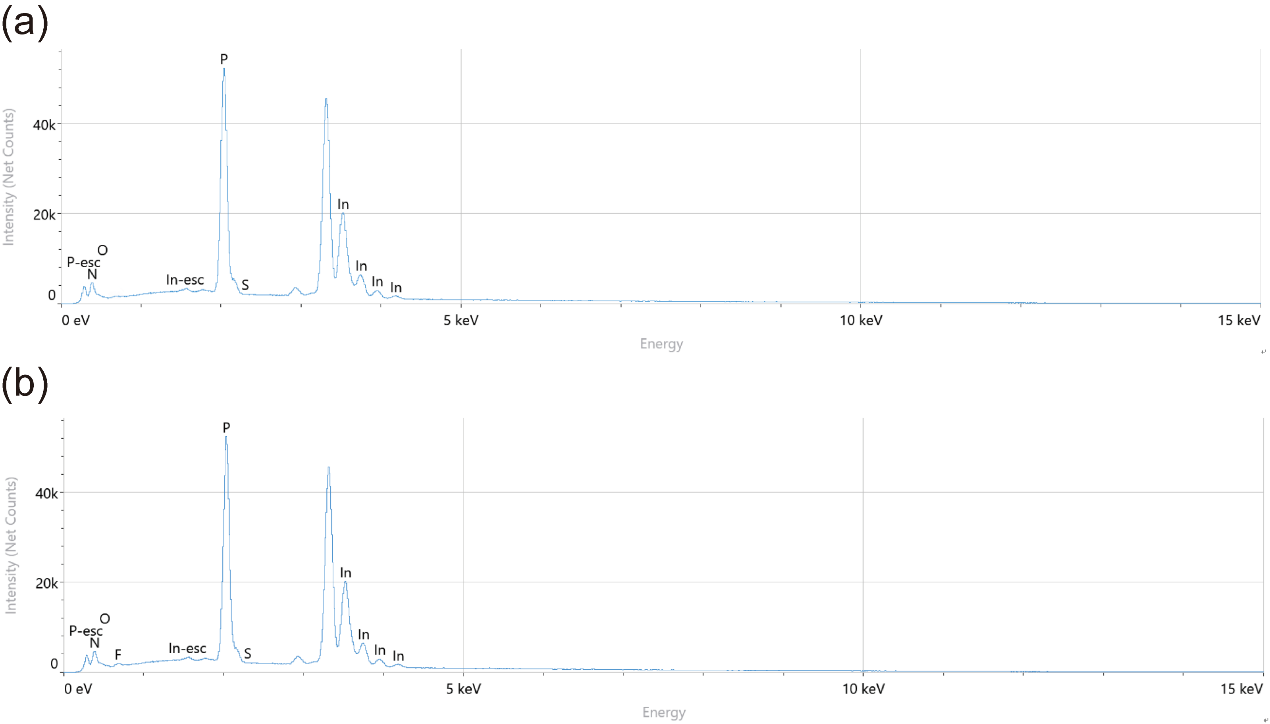


Fig. S21. EDS spectra of InP wafers: (a) untreated, and (b) after soaking in NH₄PF₆.


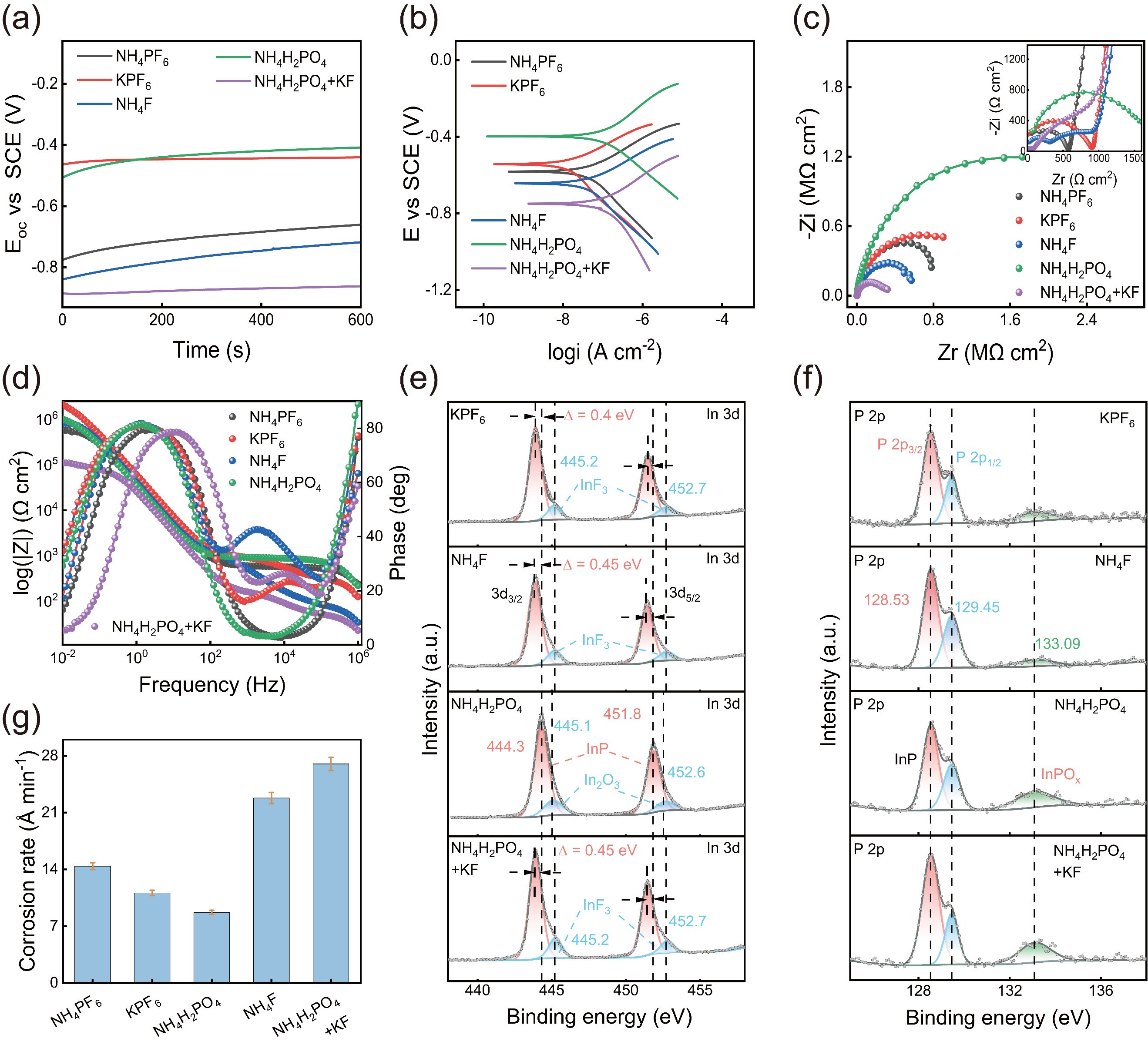


**Fig. S22.** Electrochemical and surface-analysis results for InP in different reagent solutions. Electrochemical measurements: (a) OCP, (b) Tafel, (c) Bode, and (d) Nyquist plots. XPS spectra: (e) In 3d and (f) P 2p. (g) Corresponding corrosion rates.





**Fig. S23.** MRR and Sq of Different Reagents.


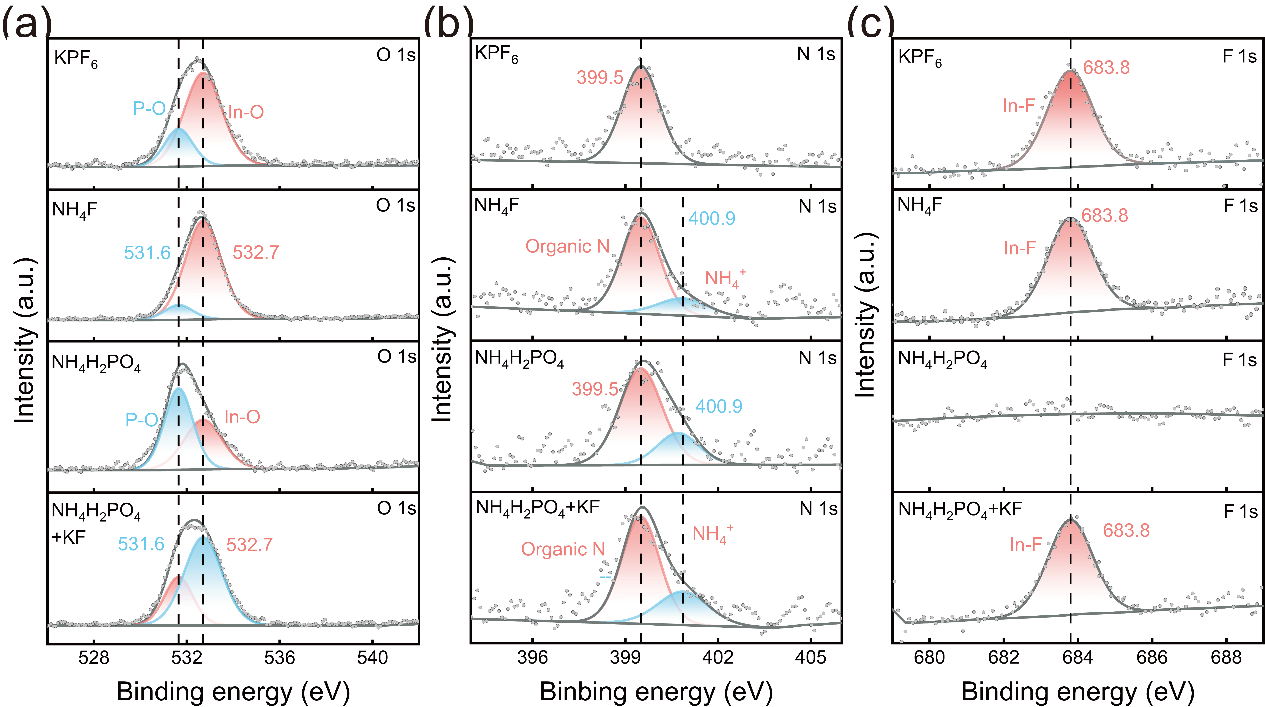


Fig. S24. XPS of KPF_6_, NH_4_F, NH_4_H_2_PO_4_ and NH_4_H_2_PO_4_+KF: (a) O 1s (b) N 1s (c) F 1s.


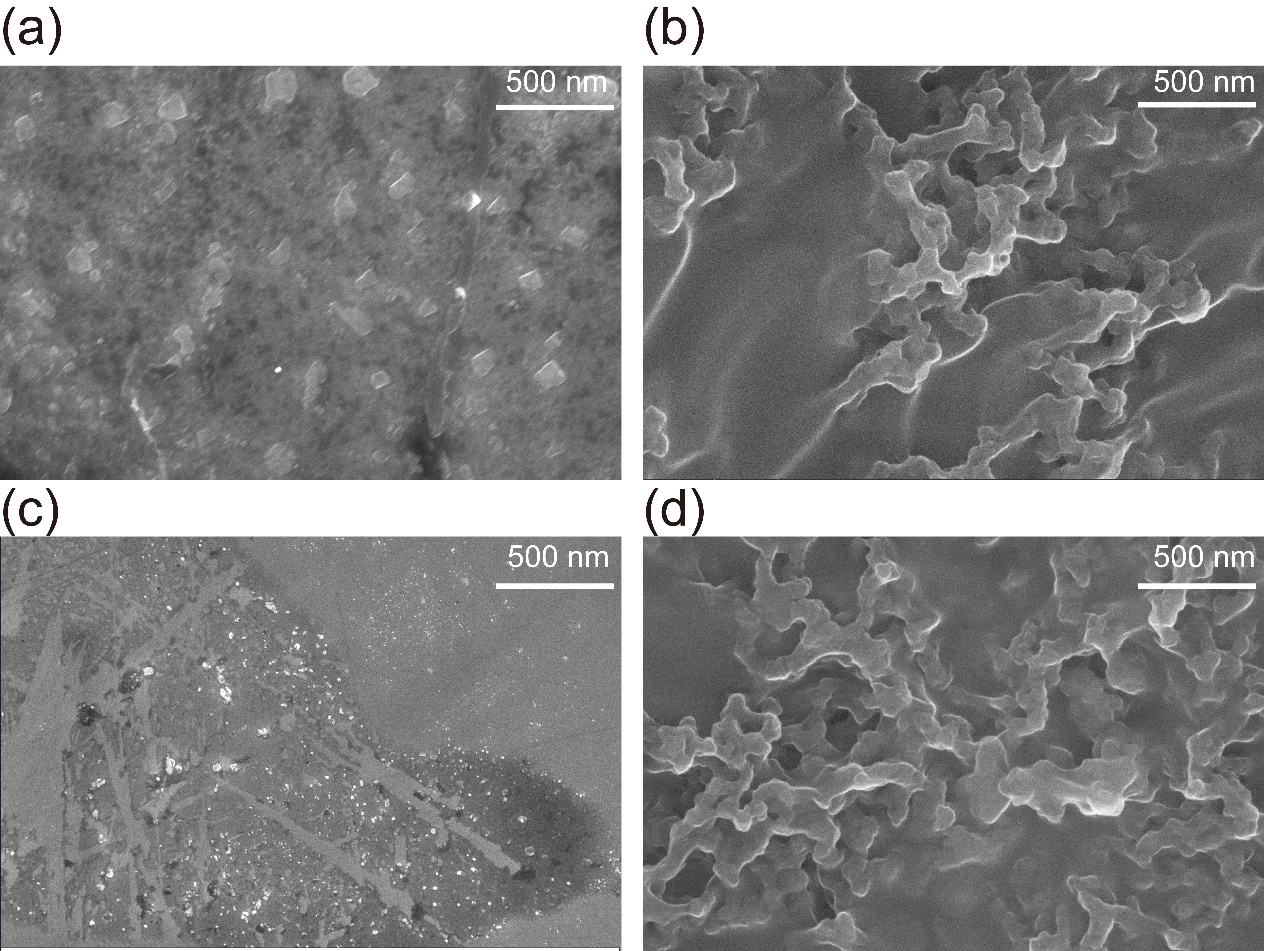


Fig. S25. SEM images of InP surfaces after soaking treatments: (a) KPF₆, (b) NH₄F, (c) NH₄H₂PO₄, and (d) NH₄H₂PO_4_+KF.


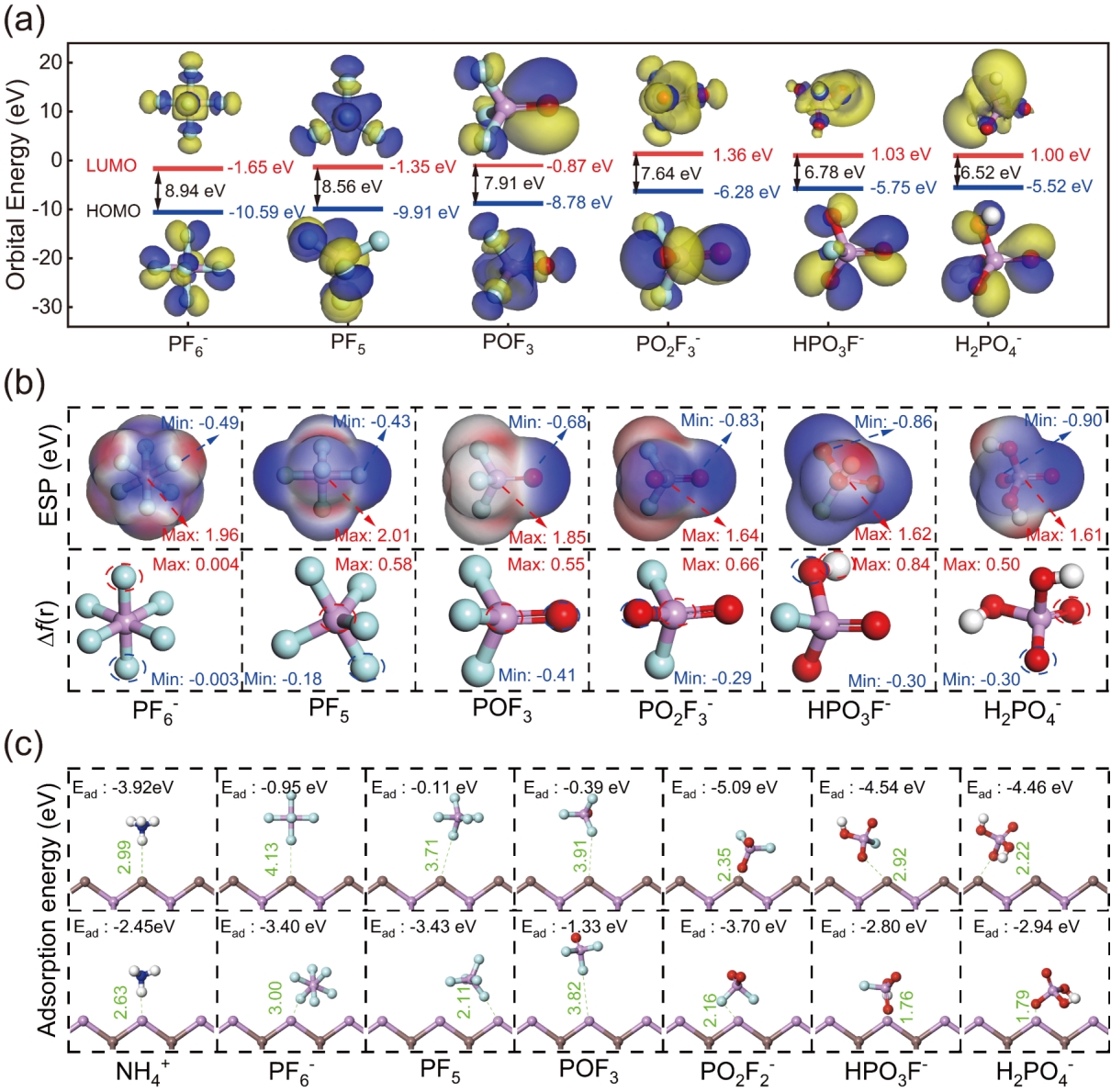


Fig. S26. Computational analysis of interactions between selected ions/molecules and InP. (a) Optimized structures and frontier-orbital (HOMO/LUMO) density distributions. (b) Electrostatic potential maps and Fukui dual descriptors. (c) Adsorption configurations on In-terminated and P-terminated InP surfaces.





Fig. S27. UV–vis integrating sphere absorption spectra of InP wafers before and after CMP polishing with NH₄PF₆.





Fig. S28. UV–vis integrating‑sphere reflection spectra of InP wafers before and after CMP polishing with NH₄PF₆.





Fig. S29. XRD patterns of InP wafers before and after CMP polishing with NH₄PF₆.





Fig. S30. Photoluminescence excitation scans of InP wafers before and after NH₄PF₆ polishing.





Fig. S31. PL spectra under different reagents at pH 3.







Fig. S32. XPS analysis of InP surfaces after CMP with NH₄PF₆ and with a commercial polishing solution: (a) In 3d spectra and (b) P 2p spectra.





Fig. S33. PLQY of commercial InP final‑polishing slurry.

**

**

Fig. S34. TRPL of InP wafers polished with NH_4_PF_6_ and with a commercial polishing slurry.

Table. S1. The effect of different concentrations of NH4PF6 on the Ecorr and Icorr of InP.

| Concentration (wt%) | Equilibrium Potential (V) | Corrosion Current (10^-8^A) |
| --- | --- | --- |
| 0.8 | -0.521 | 3.557 |
| 0.9 | -0.574 | 4.495 |
| 1.0 | -0.583 | 4.747 |
| 1.1 | -0.590 | 5.207 |
| 1.2 | -0.593 | 5.403 |
| 2 | -0.622 | 5.878 |
| 3 | -0.716 | 6.403 |
| 4 | -0.583 | 5.495 |
| 5 | -0.58 | 5.062 |

Table. S2. Impedance parameters of InP obtained by EEC fitting.

| Concentration (wt%) | Rs (Ω) | R_f_ (KΩ) | CPE_f_ (uf) | R_ct_ (KΩ) | CPE_ct_ (uf) | W(Ω·S^(-1/2)) |
| --- | --- | --- | --- | --- | --- | --- |
| 0.8 | 8.25 | 1.6 | 0.25 | 3.5 | 0.12 | 1.4 |
| 0.9 | 6.81 | 1.8 | 0.28 | 3.2 | 0.13 | 2.8 |
| 1.0 | 5.62 | 1.9 | 0.34 | 2.8 | 0.15 | 3.1 |
| 1.1 | 4.93 | 2.10 | 0.35 | 2.8 | 0.18 | 4.7 |
| 1.2 | 3.80 | 2.50 | 0.40 | 1.9 | 0.20 | 5.3 |

Table. S3. Electrical conductivity of NH_4_PF_6_ solutions at different concentrations.

| Concentration (wt%) | σ (S/m) |
| --- | --- |
| 0.8 | 7.32 |
| 0.9 | 7.05 |
| 1.0 | 7.85 |
| 1.1 | 8.65 |
| 1.2 | 9.58 |

Table. S4. Effects of different chemical reagents on the *E*_cor_r and *I*_corr_ of InP.

| Reagent | Equilibrium Potential (V) | Corrosion Current (10^-8^A) |
| --- | --- | --- |
| NH_4_PF_6_ | -0.583 | 4.747 |
| KPF_6_ | -0.545 | 4.495 |
| NH_4_F | -0.641 | 5.044 |
| NH_4_H_2_PO_4_ | -0.397 | 3.216 |
| NH_4_H_2_PO_4_+KF | -0.747 | 6.272 |

Table. S5. Impedance parameters of InP obtained by EEC fitting.

| Reagent | R_s_ (Ω) | R_f_ (KΩ) | CPE_f_ (uf) | R_ct_ (KΩ) | CPE_ct_ (uf) | W (Ω·S^(-1/2)) |
| --- | --- | --- | --- | --- | --- | --- |
| NH_4_PF_6_ | 5.62 | 1.9 | 0.34 | 2.8 | 0.15 | 3.1 |
| KPF_6_ | 6.8 | 1.8 | 0.045 | 2.8 | 32 | 2.8 |
| NH_4_F | 5.6 | 0.65 | 0.082 | 1.9 | 0.63 | 5.2 |
| NH_4_H_2_PO_4_+6KF | 4.3 | 0.98 | 0.96 | 48.7 | 1.21 | 1.43 |
| NH_4_H_2_PO_4_ | 4.6 | 0.22 | 0.35 | 1.1 | 1.8 | 3.1 |

Table. S6. Peak spectral parameters of PL spectra.

| Parameter | Blank | 0.9% | 1.0% | 1.1% |
| --- | --- | --- | --- | --- |
| E_g_(eV) | 1.333 | 1.333 | 1.332 | 1.333 |
| Peak intensity | 9684.21 | 114419.04 | 14964.60 | 14068.44 |
| FWHM (nm) | 50.7 | 49.7 | 47.3 | 50.2 |
| Stokes shift (eV) | 0.11 | - | 0.07 | - |
